# Upgrading a Piped Water Supply from Intermittent to Continuous Delivery and Association with Waterborne Illness: A Matched Cohort Study in Urban India

**DOI:** 10.1371/journal.pmed.1001892

**Published:** 2015-10-27

**Authors:** Ayse Ercumen, Benjamin F. Arnold, Emily Kumpel, Zachary Burt, Isha Ray, Kara Nelson, John M. Colford

**Affiliations:** 1 Division of Epidemiology, School of Public Health, University of California, Berkeley, Berkeley, California, United States of America; 2 Civil and Environmental Engineering, University of California, Berkeley, Berkeley, California, United States of America; 3 Aquaya Institute, Nairobi, Kenya; 4 Energy and Resources Group, University of California, Berkeley, Berkeley, California, United States of America; University of North Carolina at Chapel Hill, UNITED STATES

## Abstract

**Background:**

Intermittent delivery of piped water can lead to waterborne illness through contamination in the pipelines or during household storage, use of unsafe water sources during intermittencies, and limited water availability for hygiene. We assessed the association between continuous versus intermittent water supply and waterborne diseases, child mortality, and weight for age in Hubli-Dharwad, India.

**Methods and Findings:**

We conducted a matched cohort study with multivariate matching to identify intermittent and continuous supply areas with comparable characteristics in Hubli-Dharwad. We followed 3,922 households in 16 neighborhoods with children <5 y old, with four longitudinal visits over 15 mo (Nov 2010–Feb 2012) to record caregiver-reported health outcomes (diarrhea, highly credible gastrointestinal illness, bloody diarrhea, typhoid fever, cholera, hepatitis, and deaths of children <2 y old) and, at the final visit, to measure weight for age for children <5 y old. We also collected caregiver-reported data on negative control outcomes (cough/cold and scrapes/bruises) to assess potential bias from residual confounding or differential measurement error.

Continuous supply had no significant overall association with diarrhea (prevalence ratio [PR] = 0.93, 95% confidence interval [CI]: 0.83–1.04, *p* = 0.19), bloody diarrhea (PR = 0.78, 95% CI: 0.60–1.01, *p* = 0.06), or weight-for-age z-scores (Δz = 0.01, 95% CI: −0.07–0.09, *p* = 0.79) in children <5 y old. In prespecified subgroup analyses by socioeconomic status, children <5 y old in lower-income continuous supply households had 37% lower prevalence of bloody diarrhea (PR = 0.63, 95% CI: 0.46–0.87, *p*-value for interaction = 0.03) than lower-income intermittent supply households; in higher-income households, there was no significant association between continuous versus intermittent supply and child diarrheal illnesses. Continuous supply areas also had 42% fewer households with ≥1 reported case of typhoid fever (cumulative incidence ratio [CIR] = 0.58, 95% CI: 0.41–0.78, *p* = 0.001) than intermittent supply areas. There was no significant association with hepatitis, cholera, or mortality of children <2 y old; however, our results were indicative of lower mortality of children <2 y old (CIR = 0.51, 95% CI: 0.22–1.07, *p* = 0.10) in continuous supply areas. The major limitations of our study were the potential for unmeasured confounding given the observational design and measurement bias from differential reporting of health symptoms given the nonblinded treatment. However, there was no significant difference in the prevalence of the negative control outcomes between study groups that would suggest undetected confounding or measurement bias.

**Conclusions:**

Continuous water supply had no significant overall association with diarrheal disease or ponderal growth in children <5 y old in Hubli-Dharwad; this might be due to point-of-use water contamination from continuing household storage and exposure to diarrheagenic pathogens through nonwaterborne routes. Continuous supply was associated with lower prevalence of dysentery in children in low-income households and lower typhoid fever incidence, suggesting that intermittently operated piped water systems are a significant transmission mechanism for *Salmonella typhi* and dysentery-causing pathogens in this urban population, despite centralized water treatment. Continuous supply was associated with reduced transmission, especially in the poorer higher-risk segments of the population.

## Editors' Summary

**Background:**

Access to a safe drinking water supply (a water source that is protected from contamination with microbes or chemicals) and to adequate sanitation facilities (improved latrines and other facilities that prevent people from coming into contact with human feces) is essential for good health. Unimproved water supplies and sanitation, together with poor hygiene, increase the transmission of waterborne diseases, many of which cause diarrhea (passing three or more loose or liquid stools a day). Notably, diarrheal diseases such as cholera and dysentery kill more than three-quarters of a million children under 5 y old every year. In 2000, therefore, world leaders set a target of reducing the proportion of the global population without access to safe drinking water and basic sanitation to half of the 1990 level by 2015 as part of Millennium Development Goal 7 (MDG7; this MDG and seven others aim to eradicate extreme poverty globally). Worldwide, according to the 2015 MDG report, 2.6 billion people have gained access to improved drinking water since 1990 (91% of the world’s population now has access to an improved drinking water supply compared to 76% in 1990), and 2.1 billion people have gained access to improved sanitation.

**Why Was This Study Done?:**

The MDG target for access to safe drinking water was met by 2010, but more than 600 million people still lack access to an improved water source. Even more people lack access to piped water, the highest category of improved supply (other improved supplies include boreholes and protected wells). Moreover, piped water is only supplied intermittently in many cities in low-income countries, which can lead to waterborne illnesses through contamination in pipelines or during household storage between supply cycles and through the use of alternative unsafe water supplies. In this matched cohort study, the researchers investigate the association between continuous versus intermittent piped water supplies and waterborne diseases, mortality (death), and weight for age (an indicator of overall health and growth) among young children in Hubli-Dharwad, an Indian conurbation where about 10% of the population has a continuous piped water supply but the rest of the population only receives piped water intermittently. A matched cohort study compares outcomes in groups of people exposed to different (nonrandomized) interventions who are matched for other characteristics that might affect the outcome to allow an unbiased comparison.

**What Did the Researchers Do and Find?:**

The researchers matched the eight areas of Hubli-Dharwad with a continuous water supply to eight comparable areas with an intermittent water supply. Key characteristics used to identify matched areas included socioeconomic status and sanitation conditions. The researchers made four visits to 3,922 households in these areas with at least one child under 5 y old over a 15-mo study period to record caregiver-reported illness among young children, child deaths, and, at the final visit, the children’s weight for age. Compared to an intermittent water supply, there was no overall association between a continuous water supply and diarrhea, bloody diarrhea (dysentery), or weight-for-age scores among children aged <5 y, death before the age of 2 y, or hepatitis and cholera in the study population. However, there was a lower occurrence of bloody diarrhea among children aged <5 y in lower-income households that had a continuous water supply than in lower-income households that had an intermittent supply, and continuous supply areas had fewer household cases of typhoid fever than intermittent supply areas.

**What Do These Findings Mean?:**

The study’s reliance on caregiver reports of illnesses among young children and potential unmeasured differences between areas with intermittent versus continuous supply may affect the accuracy of these findings. Nevertheless, the study data suggest that these sources of bias are unlikely. These findings suggest that, compared to having an intermittent water supply, having a continuous water supply was not associated with diarrheal disease or growth (judged by weight) among children aged < 5 y in Hubli-Dharwad. That is, having a continuous piped water supply was not associated with a reduction in waterborne diseases overall among young children in this setting. This finding could reflect contamination of water during continued water storage in households with a continuous supply (it takes time to change old habits) and/or exposure to organisms (pathogens) that cause diarrhea through nonwaterborne routes. Importantly, however, the finding that a continuous supply was associated with a reduction in typhoid fever and more severe forms of waterborne illness among children in lower-income households suggests that intermittently operated water systems can serve as a transmission route for waterborne pathogens in urban populations despite centralized treatment of water to make it safe to drink.

**Additional Information:**

This list of resources contains links that can be accessed when viewing the PDF on a device or via the online version of the article at http://dx.doi.org/10.1371/journal.pmed.1001892.

- This study is further discussed in a *PLOS Medicine* Perspective by Clarissa Brocklehurst and Tom Slaymaker
- A *PLOS Medicine* Collection on water and sanitation is available
- The World Health Organization (WHO) provides information on water, sanitation and health (in several languages), including information on waterborne diseases and on drinking water quality; it also provides detailed information on diarrhea
- The WHO/United Nations Children's Fund (UNICEF) Joint Monitoring Programme for Water Supply and Sanitation (JMP) is the official United Nations mechanism tasked with monitoring progress toward MDG7, Target 7B; the JMP Update Report 2015 is available online (key facts are available in several languages through the JMP website)
- The children’s charity UNICEF, which protects the rights of children and young people around the world, provides information on water, sanitation and health and on diarrhea (in several languages)
- The nongovernmental organization Practical Action provides information and approaches and technologies for improving urban water supplies

## Introduction

The Joint Monitoring Programme for Water Supply and Sanitation (JMP) defines piped water as the highest category for water access. However, though widely considered the gold standard, the presence of a piped connection gives little information about the quality, quantity, and frequency of water delivery [1]. Piped water is supplied intermittently in the vast majority of cities in low-income countries [2], with segments of the distribution network supplied with water on a rotating basis for a limited number of hours at a time.

Intermittent water provision through piped networks can lead to waterborne disease risk through contamination in nonpressurized pipes, recontamination during household storage between supply cycles, use of unsafe alternative water sources, or limited water availability for hygiene. There are few studies to date on the health impact of intermittent delivery of piped water; short-term intermittencies in supply in otherwise continuously operated systems [3–6] and intermittent supply [7,8] have been associated with waterborne illness, with longer intermittencies in service typically associated with increased risk of illness [9,10].

The majority of the evidence on the health impact of intermittently operated water supplies comes from cross-sectional studies [11]. The engineering requirements to upgrade a piped system from intermittent to continuous supply mean that system improvements must be large scale, making randomized trials to study the health effects of such programs infeasible. Matched cohort designs provide a rigorous alternative in these circumstances, ensuring similar distribution of observable covariates between an intervention and comparison group, which reduces the need to rely on statistical adjustment for unbiased inference (similar to a randomized trial) [12,13]. We conducted a prospective, matched cohort study in Hubli-Dharwad, Karnataka, India, to measure the association between waterborne illness and a large program that upgraded water delivery service to continuous supply in parts of the city. To our knowledge, this study provides the first rigorous evidence for an association between continuous versus intermittent water supply and waterborne diseases and child ponderal growth.

## Methods

### Ethics

The study protocol was reviewed and approved by the Institutional Review Board of University of California, Berkeley. Primary caregivers of children <5 y old were enrolled in the study if they provided oral informed consent; written consent was not sought (with permission from IRB), as literacy rates were low among females in low-income neighborhoods and limiting enrollment to literate females would have hampered study validity. Oral consent was obtained by field staff by reading out the IRB-approved consent script to eligible participants, and a list of the households that provided oral consent to participate was maintained by the field supervisors.

### Study Setting

Hubli-Dharwad is a twin city located in Karnataka, India, with a total population close to one million [14]. In 2007–2008, approximately 10% of Hubli-Dharwad (81,000 consumers) was upgraded to receive continuous water supply through a World Bank-funded pilot project. The upgrade also included complete pipe replacement in the distribution system, new customer connections, metering, volumetric tariffs, and removal of public standpipes. The rest of the city continued to receive water intermittently, for a few hours every 5 d on average [15]. About half of the households with continuous supply in our study experienced at least one interruption in water service during the 15-mo study period; of these, 42% lasted 1–6 h, and 45% lasted 6–24 h [15].

The municipal water source for Hubli-Dharwad is centrally treated surface water from two treatment plants (Amminbhavi and Kanvihonnapur), which provide conventional water treatment with coagulation, filtration, and chlorination. During our study, the intermittent supply zones received water from both plants, while the continuous supply zones were only served by the Amminbhavi plant. However, monthly samples collected from both water treatment plants and daily samples collected from service reservoirs between November 2010 and October 2011 showed no significant differences in water quality indicators (turbidity, free chlorine residual and total coliform, and *Escherichia coli* counts) between service reservoirs supplied by the two plants, suggesting similar water quality prior to distribution system entry in intermittent and continuous supply zones [15]. Further details about Hubli-Dharwad’s water treatment and distribution systems and water quality in the distribution network are described elsewhere [15].

### Selection of Study Areas

Continuous supply was implemented in eight out of 67 city wards (i.e., administrative units of the city, typically separated from each other by boundaries such as main roads). The eight wards were selected by the Karnataka Urban Infrastructure Development and Finance Corporation (KUIDFC) based on it being possible to hydraulically isolate the ward’s pipe segment and on the ward being socioeconomically representative of Hubli-Dharwad [16]. As KUIDFC selected the continuous supply wards nonrandomly, we used a genetic matching algorithm to identify eight intermittent supply wards in Hubli-Dharwad that were comparable to the eight selected continuous supply wards on key characteristics. Genetic matching is a multivariate matching approach that uses an evolutionary search algorithm to efficiently find a set of matched pairs of treatment and control units that maximize the balance between the groups on observed characteristics (see S1 Text for details) [17]. Using an external preintervention dataset from 15,400 Hubli-Dharwad residents [18], we matched wards on socioeconomic indicators (percentages of *pukka* [concrete or reinforced cement concrete], low-income, one-room, and self-identified slum households, and illiterate females), water and sanitation conditions (percentages of households with own tap, receiving water less often than every 5 d, with own latrine, and with a designated garbage disposal location provided by the municipality and garbage collection service) and monthly household health expenses.

### Participant Selection and Enrollment

We divided each ward into socioeconomically homogeneous sampling segments separated by barriers (e.g., main roads or fields). We recruited participants from each segment in proportion to its geographical size and population density for a representative sample. In each segment, field staff started recruitment from the street closest to a selected landmark (e.g., a temple or a bus stop) and systematically approached every household until they reached the target number of participants per segment. The field team enrolled households with at least one child <5 y old and visited participants three additional times, for a total of four visits per household between November 2010 and February 2012.

### Outcome Definition and Measurement

Our primary outcome was the caregiver-reported 7-d prevalence of diarrhea in children <5 y old (i.e., occurrence of a diarrhea episode in the 7 d preceding the interview). Secondary outcomes included 7-d prevalence of blood/mucus in stool, highly credible gastrointestinal illness (HCGI), and weight for age in children aged <5 y, death of a child aged <2 y in the household, and reported incidence of typhoid fever, hepatitis, or cholera in any household member since upgrading to continuous supply in 2007–2008. We defined diarrhea as ≥3 loose stools in any 24-h period and HCGI as the occurrence of at least one episode of liquid diarrhea, soft diarrhea with abdominal cramps, vomiting, or nausea with abdominal cramps [19]. The incidence of typhoid fever, hepatitis, and cholera was recorded as reported by the respondent; diagnosis for these is typically made in local clinics in our study setting and is often symptom based without laboratory confirmation.

We specified caregiver-reported cough/cold (combined) and scrapes/bruises (combined) in children aged <5 y as negative control outcomes [20]. These symptoms should not be impacted by continuous supply; any observed association would therefore allow us to detect bias due to residual confounding from unmeasured covariates or differential measurement error from biased reporting of symptoms due to the nonblinded nature of the upgrade to continuous supply. To prevent differential probing and reporting, the data collectors and respondents were not informed that these symptoms were intended as negative control outcomes (as opposed to study outcomes of interest).

**Fig 1:**
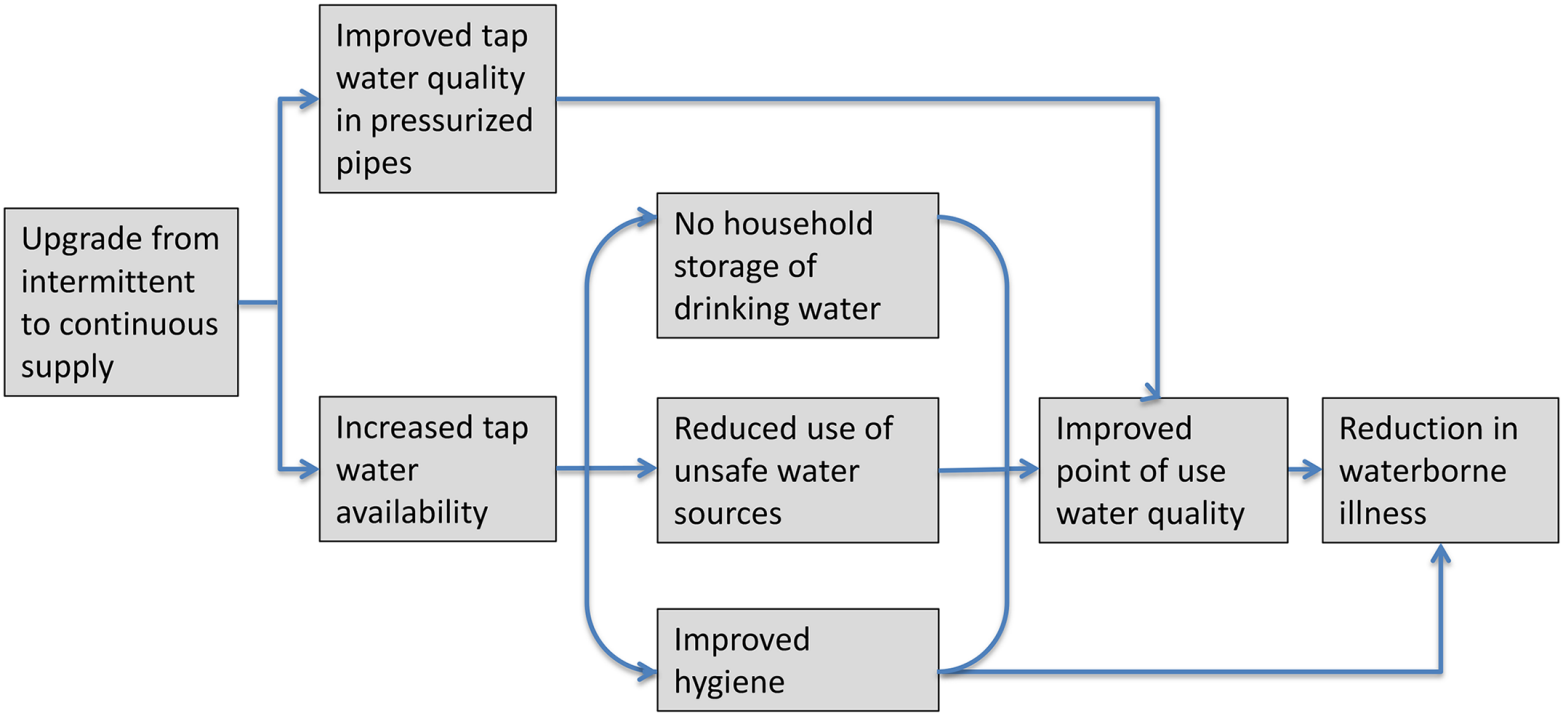
Causal chain between upgrading from intermittent to continuous water supply and reduction in waterborne illness. Upgrading from intermittent to continuous water supply is expected to be associated with reduced waterborne illness through improved tap water quality and increased water availability, which, in turn, is expected to eliminate household storage of drinking water, reduce water use from unsafe nonmunicipal sources, and improve hygiene practices.

All symptoms were recorded through a structured questionnaire administered to the primary caregiver. The 7-d prevalence of child gastrointestinal symptoms (diarrhea, HCGI, and bloody diarrhea) was ascertained during each household visit; the 3-y cumulative incidence of typhoid fever, cholera, hepatitis, and deaths of children aged <2 y was ascertained during the third visit to the households. During the final data collection round, field staff measured the weight of children aged <5 y with infant scales (Tanita 1380, 0.1 kg accuracy); measurements were taken in duplicate following standard protocols [21]. The field team also conducted structured questionnaires and spot check observations on participants’ water handling practices and collected water samples from household taps and storage containers for microbiological analysis (details described in [15]) to assess water quality, quantity, and water-related behaviors as intermediate outcomes (Fig 1).

### Statistical Methods

#### Sample size

We assumed 10% diarrhea prevalence in the intermittent supply group, an intraclass correlation coefficient (ICC) of 0.02 for households within a ward [22], 0.1 for children within a household, and 0.1 for repeated observations within a child. Assuming 1.4 children aged <5 y per household and 10% dropout, we calculated that 125 households/ward would provide 80% power to detect a 30% (three percentage-point) difference in diarrhea associated with continuous supply with a one-sided α of 0.05. We specified household socioeconomic status and rainfall a priori as potential effect modifiers; we enrolled 250 households/ward to have power for prespecified subgroup analyses for high- versus low-income households and wet versus dry weather.

#### Statistical analysis

We hypothesized that areas of Hubli-Dharwad with continuous water supply would have lower prevalence of child gastrointestinal illness, lower incidence of typhoid fever, hepatitis, and cholera, lower mortality of children <2 y old, and better child growth outcomes than the areas with intermittent supply. We prespecified to compare these outcomes between the two study groups using generalized linear models and the Wilcoxon rank-sum permutation test. All analyses were specified before the data were collected, and the analyses presented here were the only analyses planned and conducted with three exceptions: we had planned to collect additional child anthropometric measures (height, head circumference, and upper arm circumference) in addition to child weight but did not measure these because of logistical constraints. Supporting Information S1 Protocol includes the study protocol.

We calculated age- and sex-specific weight-for-age z-scores using the 2006 World Health Organization (WHO) child growth standards (zscore06, Stata 12.1). We calculated crude and adjusted measures of association for the waterborne symptoms and weight-for-age z-scores using generalized linear models and estimated CIs with bootstrapping stratified by ward and clustered by household for child-level symptoms (see S1 Text for details). For all study outcomes, we also conducted permutation tests using ward-level means and a Wilcoxon rank-sum test statistic to nonparametrically compare study groups (see S1 Text for details) [23,24]. To assess spillovers due to water sharing and/or waterborne illness transmission, we recalculated measures of association excluding households on the immediate intermittent–continuous supply boundary, defined as continuous supply areas that were not separated from neighboring intermittent supply areas by a major barrier (e.g., a main road or railway tracks) and vice versa.

We investigated effect modification by socioeconomic status and rainfall by including interaction terms in the regression models. We classified households as above- versus below-median wealth using an asset index created with principal components analysis from reported assets and observed housing materials (see S1 Text for details) [25]. We classified household visits as wet versus dry weather based on whether a local weather station had measured rain in the previous 10 d; this window allowed for the typical incubation period for most bacterial and viral diarrheagenic pathogens prior to our 7-d recall period.

To assess any bias from missing outcome data due to attrition, we compared the rate of attrition between study groups, preintervention characteristics of households that were lost to follow-up versus those that completed the study, as well as the balance of covariates between study groups both at enrollment (among all households) and at the end of the study (among the households that remained in the study). We conducted a complete-case analysis; all observed data for a given child prior to leaving the study were used in the analysis, in accordance with the assumption that outcome data were missing completely at random [26]. We also conducted an inverse probability of censoring-weighted analysis to obtain measures of association for the original enrolled population as a robustness check (see S1 Text for details) [27]. Supporting Information S1 Table includes a Strengthening the Reporting of Observational Studies in Epidemiology (STROBE) checklist.

## Results

### Characteristics of Study Wards

**Fig 2:**
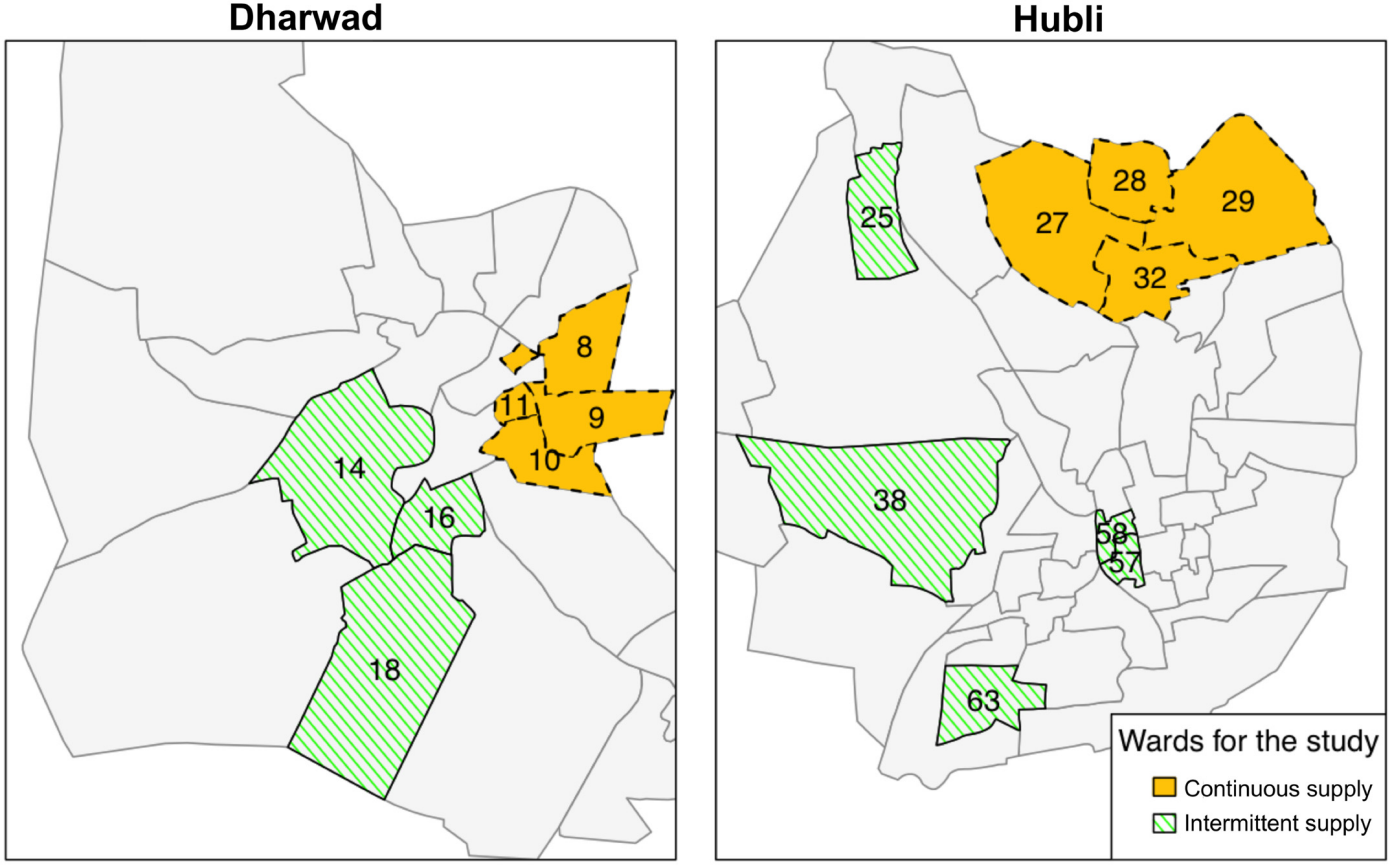
Study wards with intermittent and continuous supply in Hubli-Dharwad. Continuous supply was implemented in eight city wards (administrative units of Hubli-Dharwad) selected by the Karnataka Urban Infrastructure Development and Finance Corporation (KUIDFC). We used a genetic matching algorithm to identify eight intermittent supply wards that were comparable to the eight selected continuous supply wards on key characteristics. The numbers in the figure show the ward numbers for the 16 wards that were selected for our study.

We matched the eight continuous supply wards with eight intermittent supply wards (Fig 2). To evaluate the performance of the match prior to our data collection, we tabulated the characteristics of the matched wards using the external preintervention dataset that was used to perform the match; matching produced study groups balanced on numerous key preintervention characteristics as measured in ward-level means and standardized differences [28] (in our case, defined as difference in ward-level means between study arms divided by ward-level standard deviation in the continuous supply arm, expressed as a percent) (Table 1). Large standardized differences (e.g., >75% or 100%) are more problematic to control for with regression models; in our dataset, out of 44 preintervention variables, matching reduced the number of variables with a standardized difference >100% from eight to two and with a standardized difference >75% from 12 to five. Overall, matching improved the balance in 26 out of 44 preintervention variables. Among the 18 variables for which the balance did not improve, the magnitude of the standardized difference remained in the <25% bracket for eight, in the 25%–75% bracket for five, and moved from <25% to slightly >25% for five (S2 Table).

**Table 1:** Comparison of ward characteristics by study group before and after matching. Source of external dataset: Socioeconomic survey of Hubli-Dharwad city [18].

	Full Set of Intermittent Supply Wards (*N* = 59)		Matched Set of Intermittent Supply Wards (*N* = 8)		Continuous Supply Wards (*N* = 8)
	Mean/%	Standardized Difference ^a^	Mean/%	Standardized Difference ^a^	Mean/%
**Demographics and socioeconomics**					
Mean number of persons per household	5.1	−68	5.0	−30	4.8
Mean number of children aged <5 y per household	1.4	−115	1.4	−85	1.3
% of illiterate females	17.7	−13	16.4	15	17.1
% of individuals working as agricultural laborer	12.0	6	12.3	2	12.4
% of non-Hindu households	26.0	−83	22.2	−40	18.7
% of scheduled caste or tribe households	14.6	−94	10.4	4	10.5
% of slum households (self-report)	22.8	10	23.7	6	25.0
% of migrant households	14.5	−63	13.1	−47	8.6
% of BPL card holder households ^b^	25.9	−8	26.7	−14	24.8
% of households with income <US$350/year	12.2	−47	11.6	−37	9.5
% of households that own their home	65.9	92	72.5	16	73.9
% of *pukka* homes ^c^	71.5	15	75.6	−11	73.9
% of one-room homes	6.3	−213	5.2	−141	2.9
% of households that have:					
Electricity	94.4	49	95.4	11	95.8
Fridge	16.0	−10	17.0	−17	14.6
Bicycle	26.9	41	30.4	15	32.4
Motorcycle	29.7	7	35.6	−33	30.6
Phone	38.4	1	40.4	-8	38.7
Radio	39.4	51	42.5	30	46.7
**Water, sanitation, and hygiene conditions**					
% of households with own tap	79.5	136	88.9	25	91.1
% of households receiving water every 5 or more d	7.7	−313	2.8	−72	1.3
% of households with own latrine	74.5	32	79.1	5	80.0
% of households served by open drain	8.4	65	12.0	55	31.3
% of households with designated garbage bin or collection at door	47.1	9	45.6	16	49.0
% of households with garbage cleared regularly by municipality	37.5	−9	37.9	−11	35.5
% of households with health expenditures >US$2/month	24.5	46	21.1	64	33.3

Abbreviations: BPL, below poverty level.

^a^ Standardized difference is the difference between ward-level means in two study arms divided by the ward-level standard deviation in continuous supply arm.

^b^ A BPL card is issued by the government based on household income.

^c^ *Pukka* refers to concrete or reinforced cement concrete.

### Characteristics of Study Participants

**Fig 3:**
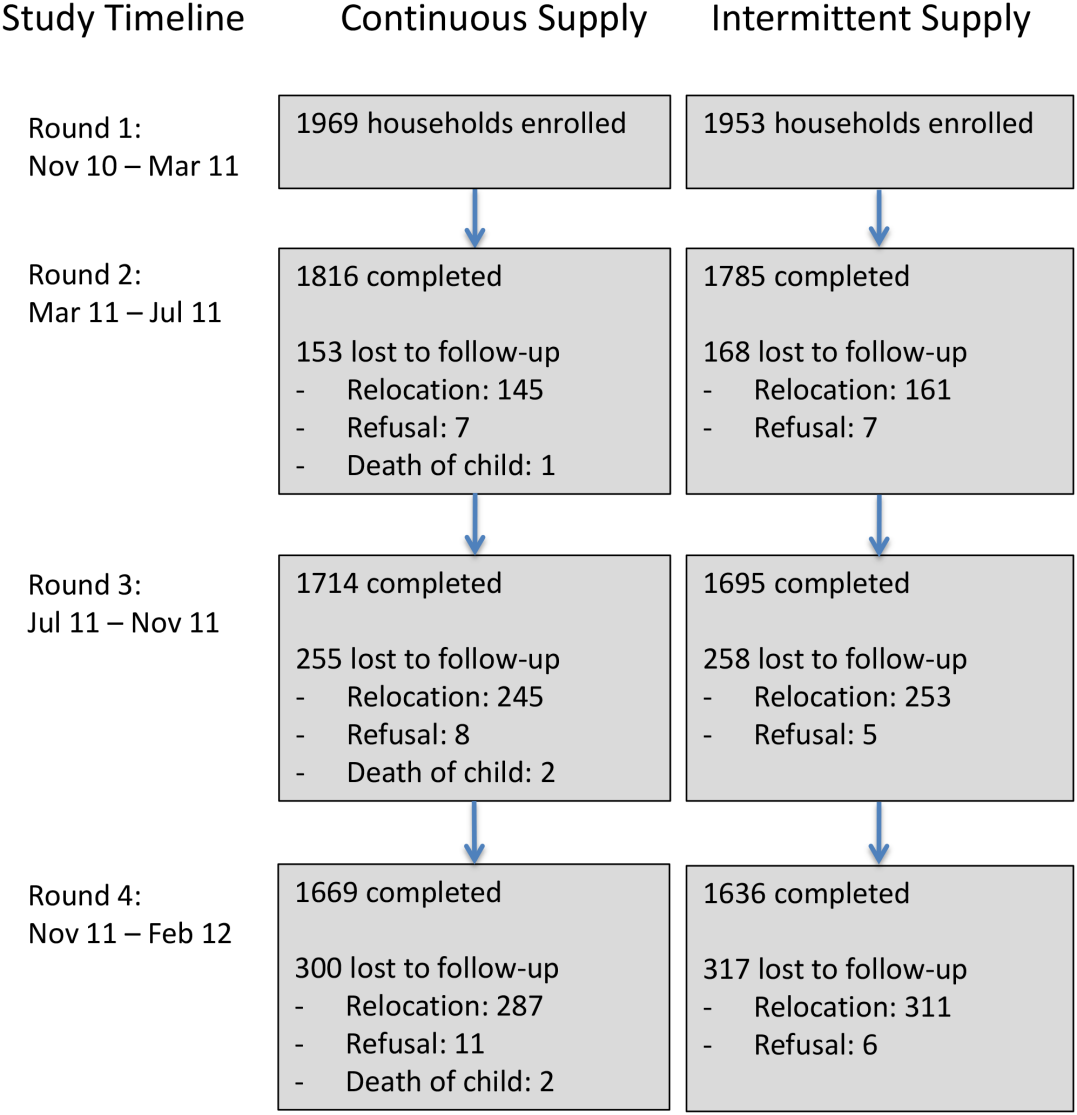
Number of households enrolled and lost to follow-up across study arms and data collection rounds. Of the 3,922 households we enrolled, 3,305 (84%) completed the study, while 617 households were lost to follow-up over the course of four rounds of data collection. The number of households lost to follow-up at each round was similar between the two study arms.

We enrolled 3,922 households with 5,420 children aged <5 y and collected postintervention data on household characteristics to re-evaluate the performance of the match based on our study data. Study arms were well balanced across a wide range of covariates (Table 2). Of enrolled households, 3,305 (84%) completed the study, while 617 households were lost to follow-up (598 relocated, 17 refused, and in two households the enrolled child died). The rate of loss to follow-up was similar in the two groups (Fig 3), suggesting nondifferential attrition by study arm, and households that completed the study remained well balanced across all covariates (Table 2). Households that were lost to follow-up (i.e., did not complete the study) were similar to those that remained in the study, except that they were less likely to be homeowners (S3 Table); this suggests that missing data were “covariate-dependent completely missing at random” [26]. We hypothesize this is because renters were more likely to relocate than those residing in their self-owned home. The loss of nonhomeowners enriched our study population in homeowners from ~65% at enrollment to ~70% at the end of the study; however, among the households that remained in the study, the proportion of homeowners remained well balanced between study groups (Table 2).

**Table 2:** Comparison of household characteristics by study group (among all households at enrollment and among households that remained at end of study).

	At Enrollment (*N* = 3922)				At End of Study (*N* = 3305)			
	Continuous Supply		Intermittent Supply		Continuous Supply		Intermittent Supply	
	*N*	Mean/%	*N*	Mean/%	*N*	Mean/%	*N*	Mean/%
**Demographics and socioeconomics**								
Mean number of persons per household	1,968	6.5	1,951	6.5	1,668	6.7	1,634	6.7
Mean number of children aged <5 y per household	1,968	1.4	1,951	1.4	1,668	1.4	1,634	1.4
Mean age of primary caregiver of children aged <5 y	1,956	26.9	1,945	27.0	1,656	27.0	1,629	27.2
Mean monthly household income (USD)	1,562	195.9	1,466	202.9	1,321	198.2	1,233	206.6
Mean number of rooms in household	1,967	2.4	1,951	2.3	1,667	2.5	1,634	2.4
% of households with:								
*Pukka* roof ^a^	1,967	44.0	1,951	45.2	1,667	44.5	1,634	45.3
*Pukka* walls ^a^	1,814	56.4	1,781	63.4	1,637	56.7	1,600	63.1
*Pukka* floor ^a^	1,814	95.1	1,781	96.5	1,637	94.9	1,600	96.6
Fridge	1,968	25.5	1,951	30.2	1,668	25.3	1,634	31.1
Motorcycle	1,968	47.4	1,951	48.9	1,668	48.3	1,634	50.4
Mobile phone	1,968	90.5	1,951	89.9	1,668	90.9	1,634	90.1
% of households owning at least one home	1,966	66.9	1,951	64.4	1,666	71.5	1,634	69.8
% of self-employed father	1,962	32.7	1,945	35.3	1,662	32.9	1,628	36.6
% of illiterate mother	1,962	8.5	1,948	10.1	1,662	8.0	1,632	10.4
% Hindu	1,967	73.1	1,951	66.0	1,667	72.5	1,634	66.8
**Water, sanitation, and hygiene indicators**								
% of households with handwashing facility:								
Inside the household	1,968	73.6	1,951	73.5	1,668	73.7	1,634	73.6
In yard	1,968	24.8	1,951	25.1	1,668	24.9	1,634	25.3
No specific place	1,968	1.6	1,951	1.4	1,668	1.4	1,634	1.1
% of households with sanitation access:								
Private latrine	1,815	91.2	1,782	91.6	1,638	91.0	1,600	91.8
Public latrine	1,815	6.0	1,782	3.6	1,638	6.1	1,600	3.7
No latrine	1,815	2.9	1,782	4.7	1,638	2.9	1,600	4.6
% of households where children aged <5 y defecate:								
In latrine or potty	1,811	64.9	1,781	65.0	1,634	65.5	1,599	65.3
In area within household compound	1,811	19.5	1,781	16.5	1,634	19.5	1,599	16.6
In area outside household compound	1,811	16.9	1,781	19.5	1,634	16.7	1,599	19.1
% of households with sewerage in vicinity:								
Underground piped sewer	1,813	78.3	1,777	74.4	1,636	78.4	1,596	74.1
Open drain or open sewage canal	1,813	71.6	1,777	75.1	1,636	71.9	1,596	75.3
% of household with garbage disposal:								
In open heap	1,814	34.5	1,781	25.9	1,637	34.3	1,600	25.2
Designated bin	1,814	43.9	1,781	45.8	1,637	44.0	1,600	46.5
Collected at the door	1,814	18.6	1,781	24.5	1,637	18.6	1,600	24.6

Abbreviations: USD, US dollars.

^a^ *Pukka* refers to concrete or reinforced cement concrete.

### Water-Related Infrastructure and Practices

We also collected postintervention data on household water infrastructure and practices. Households in continuous supply wards were more likely to collect water from their own tap and less likely to use borewells (Table 3). Tap water quality was significantly better under continuous supply, with <1% of samples positive for *E*. *coli* and thus not meeting WHO drinking water quality guidelines versus 32% under intermittent supply [15,29]. However, participants with continuous supply continued to store drinking water (Table 3), and contamination during storage was common; 13% of stored water samples were positive for *E*. *coli* in continuous supply households versus 34% in intermittent supply households [15]. Drinking water treatment was equally (in)frequent in both groups (Table 3). Municipal water consumption was higher under continuous water supply and met the often-cited recommendation of 50 liters per capita per day, while approximately half of intermittent supply households fell short of this target [30,31]. Despite the difference in municipal water consumption, there was no difference in observed water availability at the handwashing facility between the two groups (Table 3). Customer satisfaction with tap water quality, quantity, and pressure showed seasonal variation, especially in intermittent supply households (S4 Table).

**Table 3:** Water infrastructure and water-related household behaviors by study arm.

	Continuous Supply		Intermittent Supply	
	*N*	%	*N*	%
**Water infrastructure and services**				
Collects municipal water from:				
Own connection	1,968	67.3	1,951	58.3
Landlord's or neighbor's connection	1,968	32.5	1,951	35.4
Public connection	1,968	0.2	1,951	6.3
Location of tap:				
Indoors within household	1,964	35.1	1,907	17.9
Outdoors within premises	1,964	61.0	1,907	69.3
Not on premises	1,964	3.3	1,907	10.9
Location of mouth of tap:				
Elevated from the ground	1,966	85.9	1,881	69.9
On the ground	1,966	13.5	1,881	17.3
Inside a tank	1,966	0.4	1,881	12.2
Customer satisfaction: ^a^				
Tap water does not smell or look dirty	7,090	72.5	7,006	59.8
Happy with tap water quality	7,166	77.4	7,066	54.4
Happy with tap water quantity	7,166	93.8	7,066	67.9
Happy with tap water pressure	7,166	90.1	7,066	53.6
**Water-related household behaviors**				
Retrieves drinking water from: ^a^				
Tap connected directly to waterline	7,008	9.5	6,896	1.6
Tap connected to overhead tank	7,008	0.7	6,896	0.2
Storage container	7,008	76.5	6,896	83.4
Commercial water treatment device	7,008	11.8	6,896	13.3
Bottled water	7,008	1.4	6,896	1.5
Treats drinking water ^a^	7,024	26.7	6,914	26.8
Collects water from other sources: ^a^				
Borewell (public or private)	7,167	5.4	7,067	37.8
Water truck	7,167	0.0	7,067	2.1
Washes vegetables:				
Outside	1,815	27.5	1,783	15.4
In kitchen	1,815	57.3	1,783	76.8
In bathroom	1,815	14.9	1,783	7.6
Washes utensils:				
Outside	1,815	71.3	1,783	78.7
In kitchen	1,815	12.8	1,783	10.7
In bathroom	1,815	15.5	1,783	9.9
Has handwashing facility with water	1,962	95.9	1,949	93.4

^a^ The *N* for these variables is higher because these questions were asked at each round.

### Waterborne Diseases and Weight for Age

Diarrhea prevalence in children aged <5 y was 8% over the study period (1,640 prevalent cases over four rounds of data collection). The prevalence of blood/mucus in stool was 2% (343 cases), and HCGI prevalence was 11% (2,293 cases). All symptoms were more prevalent in children in below-median wealth households. The mean weight-for-age z-score for <5-y-old children was −1.58 (standard deviation [SD] = 1.13). In the three years since upgrading to continuous supply, 5% (161) of households had at least one case of typhoid fever, 3% (105) had hepatitis, 0.3% (10) had cholera, and 1% (32) lost a child aged <2 y, as reported during the third round of data collection.

We found no significant overall association between continuous versus intermittent supply and diarrhea (PR = 0.93, 95% CI: 0.83–1.04, *p* = 0.19, permutation test *p*-value = 0.94), bloody diarrhea (PR = 0.78, 95% CI: 0.60–1.01, *p* = 0.06, permutation test *p*-value = 0.25), HCGI (PR = 1.01, 95% CI: 0.92–1.11, *p* = 0.78, permutation test *p*-value = 0.78) or weight for age (Δz = 0.01, 95% CI: −0.07–0.09, *p* = 0.79, permutation test *p*-value = 0.67) in <5-y-old children (Tables 4 and 5). Subgroup analyses by socioeconomic status showed an association with bloody diarrhea in low-income households; <5-y-old children in below-median wealth continuous supply households had 37% lower prevalence of blood or mucus in stool (PR = 0.63, 95% CI: 0.46–0.87, *p*-value for interaction = 0.03) compared to below-median wealth intermittent supply households. There was no association between continuous versus intermittent supply and child waterborne symptoms in above-median wealth households (Table 4). The impact of rainfall on the measures of association was inconclusive (Table 4).

**Table 4:** Seven-day prevalence of child diarrheal illness (children aged <5 y), aggregated over four rounds of data collection.

	Intermittent Supply		Continuous Supply							
	N	P	*N*	P	PR	(95% CI) ^a^	Adjusted PR ^b^	95% CI ^a^	Regression *p*-value	Permutation test *p*-value ^c^
**Main analysis**										
HCGI	10,000	11.3	10,035	11.5	1.02	(0.93–1.11)	1.01	(0.92–1.11)	0.78	0.78
Diarrhea (primary outcome)	10,019	8.4	10,054	7.9	0.94	(0.84–1.05)	0.93	(0.83–1.04)	0.19	0.94
Blood or mucus in stool	10,016	1.9	10,052	1.5	0.81	(0.65–1.02)	0.78	(0.60–1.01)	0.06	0.25
**Subgroup analysis by wealth**									Interaction *p*-value ^d^	
**Above median wealth**										
HCGI	5,034	10.1	5,026	10.4	1.03	(0.90–1.18)	1.04	(0.91–1.19)	0.59	
Diarrhea	5,043	7.0	5,037	6.9	0.98	(0.83–1.17)	0.98	(0.84–1.16)	0.36	
Blood or mucus in stool	5,041	1.2	5,036	1.4	1.11	(0.77–1.58)	1.08	(0.73–1.63)	0.03	
**Below median wealth**										
HCGI	4,960	12.6	4,983	12.6	1.00	(0.88–1.13)	0.99	(0.86–1.13)	--	
Diarrhea	4,970	9.8	4,991	8.8	0.90	(0.78–1.04)	0.89	(0.76–1.04)	--	
Blood or mucus in stool	4,969	2.5	4,990	1.6	0.64	(0.47–0.86)	0.63	(0.46–0.87)	--	
Subgroup analysis by rainfall									Interaction *p*-value ^d^	
**Dry period (>10 d after rain)**										
HCGI	4,276	10.7	4,337	11.3	1.05	(0.93–1.20)	1.05	(0.92–1.21)	0.42	
Diarrhea	4,284	8.3	4,343	8.4	1.01	(0.87–1.18)	1.01	(0.87–1.18)	0.14	
Blood or mucus in stool	4,282	2.2	4,342	1.5	0.70	(0.50–0.95)	0.69	(0.49–0.98)	0.30	
**Wet period (<10 d after rain)**										
HCGI	5,724	11.8	5,698	11.7	0.99	(0.89–1.11)	0.99	(0.88–1.11)	--	
Diarrhea	5,735	8.5	5,711	7.6	0.89	(0.77–1.02)	0.87	(0.75–1.00)	--	
Blood or mucus in stool	5,734	1.6	5,710	1.5	0.93	(0.68–1.25)	0.87	(0.62–1.22)	--	

Abbreviations: P, prevalence (%); PR, prevalence ratio; CI, confidence interval; HCGI, highly credible gastrointestinal illness.

^a^ CIs obtained by bootstrapping within strata of wards with clustering at household level.

^b^ Adjusted for child age, child sex, season, household socioeconomic status, religion, handwashing infrastructure, latrine ownership, sewerage, and garbage disposal; we only included covariates in the adjusted models that could not plausibly be impacted by the continuous supply intervention.

^c^ *p*-value from Wilcoxon rank-sum permutation test; the permutation test is conservative relative to the CIs around the PR because it tests the null hypothesis that the two groups have the same distribution as opposed to the null hypothesis of no effect on average.

^d^ *p*-value for interaction from generalized linear model with interaction terms.

**Table 5:** Weight-for-age z-score (children aged <5 y), ascertained at last round of data collection.

	Intermittent Supply		Continuous Supply							
	*N*	Mean z-score (SD)	*N*	Mean z-score (SD)	Δz	95% CI ^a^	Adjusted Δz ^b^	95% CI ^a^	Regression *p*-value	Permutation test *p*-value ^c^
All observations ^d^	1,765	−1.59 (1.14)	1,811	−1.58 (1.12)	0.01	(−0.07–0.09)	0.01	(−0.07–0.09)	0.79	0.67
Subgroup analysis by wealth									Interaction *p*-value ^e^	
Above median wealth	934	−1.48 (1.20)	935	−1.48 (1.15)	−0.01	(−0.12–0.11)	0.01	(−0.11–0.12)	0.95	--
Below median wealth	829	−1.71 (1.07)	870	−1.68 (1.09)	0.03	(−0.07–0.14)	0.01	(−0.10–0.13)	--	--
Subgroup analysis by rainfall									Interaction *p*-value ^e^	
Dry period (>10 d after rain)	1,610	−1.59 (1.14)	1,596	−1.60 (1.13)	−0.01	(−0.09–0.08)	−0.01	(−0.10–0.07)	0.10	--
Wet period (<10 d after rain)	155	−1.57 (1.19)	215	−1.39 (1.03)	0.17	(−0.08–0.41)	0.21	(−0.03–0.45)	--	--

Abbreviations: CI, confidence interval; SD, standard deviation.

^a^ CIs obtained by bootstrapping within strata of wards with clustering at household level.

^b^ Adjusted for household socioeconomic status, religion, handwashing infrastructure, latrine ownership, sewerage, and garbage disposal; we only included covariates in the adjusted models that could not plausibly be impacted by the continuous supply intervention.

^c^ *p*-value from Wilcoxon rank-sum permutation test; the permutation test is conservative relative to the CIs around the PR because it tests the null hypothesis that the two groups have the same distribution as opposed to the null hypothesis of no effect on average.

^d^ We excluded children with z-scores >5 or <−5 from the analysis.

^e^ *p*-value for interaction from generalized linear model with interaction terms.

In continuous supply wards, 42% fewer households had at least one reported case of typhoid fever compared to intermittent supply wards (CIR = 0.58, 95% CI: 0.41–0.78, *p* = 0.001, permutation test *p*-value = 0.43, Table 6). There was no significant association between continuous versus intermittent supply and cholera (CIR = 1.48, 95% CI: 0.37–6.92, *p* = 0.58, permutation test *p*-value = 0.69), hepatitis (CIR = 1.13, 95% CI: 0.76–1.73, *p* = 0.54, permutation test *p*-value = 0.67), or <2-y-old child deaths (CIR = 0.51, 95% CI: 0.22–1.07, *p* = 0.10, permutation test *p*-value = 0.16) (Table 6). While our findings were indicative of lower <2-y-old mortality associated with continuous versus intermittent supply, we could not rule out chance as an explanation of this difference because of the small number of deaths (*N* = 32).

**Table 6:** Waterborne disease incidence and child mortality (children aged <2 y) since implementation of continuous supply, ascertained at the third round of data collection.

	Intermittent Supply			Continuous Supply								
	*N*	HHs with case	I ^a^	*N*	HHs with case	I ^a^	CIR	95% CI ^b^	Adjusted CIR ^c^	95% CI ^b^	Regression *p*-value	Permutation test *p*-value ^d^
Typhoid	1,690	103	60.9	1,711	58	33.9	0.56	(0.40–0.76)	0.58	(0.41–0.78)	0.001	0.43
Cholera	1,691	4	2.4	1,711	6	3.5	1.48	(0.37–6.92)	-- ^e^	--	0.59	0.69
Hepatitis	1,690	46	27.2	1,711	59	34.5	1.27	(0.87–1.87)	1.13	(0.76–1.73)	0.54	0.67
<2-y-old child death	1,695	20	11.8	1,713	12	7.0	0.59	(0.26–1.19)	0.51	(0.22–1.07)	0.10	0.16

Abbreviations: HH, household; I: incidence; CIR, cumulative incidence ratio; CI, confidence interval.

^a^ Households with at least one reported case (per 1,000 households) since implementation of continuous supply.

^b^ CIs obtained by bootstrapping within strata of wards.

^c^ Adjusted for household socioeconomic status, religion, handwashing infrastructure, latrine ownership, sewerage, and garbage disposal; we only included covariates in the adjusted models that could not plausibly be impacted by the continuous supply intervention.

^d^ *p*-value from Wilcoxon rank-sum permutation test; the permutation test is conservative relative to the CIs around the CIR because it tests the null hypothesis that the two groups have the same distribution as opposed to the null hypothesis of no effect on average.

^e^ Adjusted CIR not calculated because of sparse data.

Crude and adjusted measures of association were similar for all outcomes (Tables 4, 5 and 6). Negative control outcomes did not differ between continuous versus intermittent supply groups (PR = 1.00, 95% CI: 0.96–1.05, *p* = 0.96, permutation test *p*-value = 1.00 for cough/cold, and PR = 1.12, 95% CI: 0.97–1.29, *p* = 0.10, permutation test *p*-value = 0.51 for scrapes/bruises), except for a higher prevalence of scrapes/bruises in the continuous supply group in the wet season (S5 Table). Excluding households on the intermittent–continuous supply boundary to eliminate any spillovers did not change the results (S6 and S7 Tables). The inverse probability of censoring-weighting analysis yielded results similar to the complete-case analysis (S8 Table).

## Discussion

### Continuous Supply and Gastrointestinal Illness

We found no significant overall association between continuous versus intermittent water supply and diarrheal illness symptoms or weight for age in <5-y-old children in Hubli-Dharwad, India (Tables 4 and 5). However, continuous supply was associated with lower prevalence of more severe forms of diarrhea among children living in the poorer households; <5-y-old children in below-median wealth continuous supply households had 37% lower bloody diarrhea prevalence (PR = 0.63, 95% CI: 0.46–0.87) compared to below-median wealth intermittent supply households (Table 4). The percentage of households with a reported case of typhoid fever in continuous supply wards was 42% lower than in intermittent supply wards (CIR = 0.58, 95% CI: 0.41–0.78) (Table 6). Two previous studies conducted in socioeconomically disadvantaged communities in Mexico and Palestine found odds ratios of 0.50 (95% CI: 0.27–0.86) and 0.65 (95% CI: 0.49–0.87), respectively, for diarrhea associated with access to continuous versus intermittent water supply [7,8]. While our study showed no significant association with general diarrheal symptoms, the magnitude of the differences we observed between study groups in the prevalence of bloody diarrhea in children in low-income households and in the incidence of typhoid fever are consistent with the findings of these studies.

### Factors Mediating the Association between Continuous Supply and Gastrointestinal Illness

Continuous supply households had better tap water quality, with <1% of samples not meeting WHO guidelines versus 32% under intermittent supply [15]. We also found higher access to municipal water and less frequent use of water from nonmunicipal sources under continuous supply (Table 3); this could be driven by the removal of public standpipes in the continuous supply areas as part of the upgrade, as well as the lessened need due to continuous water availability at the tap. We observed no difference in water availability for hygiene between study groups (Table 3); again, this could be explained by intermittent supply households augmenting their municipal supply from alternative sources such as borewells and water trucks.

Notably, households continued to store drinking water even after the upgrade to continuous supply [32]. Reasons for this might include habit, interruptions in service (half of continuous supply respondents experienced at least one intermittency over the 15-mo study period, mostly ranging 1–24 h), and fear of longer interruptions, as well as convenience (households often have their tap connections at the compound entrance, making it inconvenient to directly use tap water and necessitating storage in the kitchen). Reliance on stored water may decline over time as users build confidence in uninterrupted water delivery; however, we note that at the time of our evaluation, continuous supply had already been in place for approximately 3 y, albeit with sporadic short-term intermittencies. Household storage of drinking water is problematic because stored water becomes contaminated with pathogens through contact with hands and fomites [33]. We found that, while stored water quality was better under continuous supply (13% not meeting guidelines versus 36% under intermittent supply), drinking water quality deteriorated in storage in both groups. It is possible that additional measures to minimize household storage and reduce point-of-use contamination during storage would enhance the health benefits from continuous supply. However, given the relatively small percentage of contaminated point-of-use water samples in continuous supply households, it is unlikely that water storage fully explains the lack of significant differences in diarrheal disease between study groups.

We note that several sanitation and hygiene-related factors might have counteracted the benefits of continuous water supply in this setting. While latrine access in our study population was high, with >90% households in both study arms having access to a private latrine, approximately 75% of respondents had an open drain or sewage canal on their street and, in 35% of households, children <5 y old were reported to openly defecate within or outside the household compound. Additionally, approximately 30% of households collected their garbage in open heaps. Roughly 30% of continuous supply households reported washing vegetables outside, and 70% reported washing utensils outside, presenting ample opportunity for fecal contamination of food, fomites, and hands and potentially offsetting the health benefits of improved water quality through continuous water supply. Pathogen exposure through nonwaterborne routes may also explain the high prevalence of diarrhea even in higher-income households with continuous water supply in this setting.

### Continuous Supply and Severe Gastrointestinal Illness in High-Risk Populations

In contrast to general diarrhea outcomes, we observed significantly lower typhoid fever incidence associated with continuous water supply in the study population. A plausible explanation for this might be that the municipal water supply is responsible for a larger proportion of typhoid transmission than for overall diarrhea, which has multiple etiologies and transmission pathways. We have evidence that improvements in urban water infrastructure in the early 1900s led to dramatic reductions in typhoid fever mortality in the United States[34]. Others have also found evidence linking typhoid to contamination in municipal water supplies in low-income country cities [35–38]. This is also consistent with previous research demonstrating higher typhoid incidence in urban versus rural populations [39].

As for bloody diarrhea, infections that lead to blood or mucus in the stool are typically caused by shigellosis, amoebic dysentery, and enterohaemorrhagic *E*. *coli*, which are transmitted through waterborne and water-washed pathways [40]; improvements in water quality and quantity through the upgrade to continuous supply would therefore plausibly reduce the prevalence of these infections. These findings also suggest that blood or mucus in stool could be used as a more specific indicator of waterborne illness than overall diarrhea symptoms with multiple etiologies. Notably, the lower prevalence of dysentery in continuous versus intermittent supply households was only observed in low-income segments of the population. While it is possible that this finding presents a false positive due to multiple subgroup analyses, our data show trends to support that this is a true causal finding rather than a standalone chance positive; as we move from the least specific symptom (HCGI) to the most specific (blood/mucus in stool), there is a qualitative trend among low-income households of progressively larger differences between intermittent and continuous supply groups.

Poorer households experiencing larger health benefits is also biologically plausible. Low-income areas in Hubli-Dharwad typically have poorer sanitation infrastructure; 80% of low-income participants in our study had open drains versus 67% of higher-income participants. The risk of pathogen intrusion into pipelines is increased when there is fecal contamination near waterlines [41]. The protective impact of continuous pressure could therefore be greater in low-income neighborhoods, where unsanitary conditions may facilitate pathogen intrusion into pipes under intermittent pressure. Household water treatment was also more prevalent among higher-income households (39% versus 14% in lower-income households). Any improvements in municipal water quality would have a bigger health impact in low-income residents who consume this water untreated. Finally, higher-income households have access to high-capacity storage tanks, while low-income households rely on small storage containers to secure water until the following supply cycle; 61% of higher-income households versus 8% of low-income households in our study used an overhead tank. The latter therefore experiences a bigger water strain during intermittencies and could benefit more from continuous supply. These findings lend credibility to larger health benefits from continuous supply in low-income populations.

### Limitations

#### Confounding from unobserved covariates

Given engineering requirements, we expect that it would be nearly impossible to randomize continuous versus intermittent water supply in urban settings. The matched cohort design is a strong alternative to randomization to construct well-balanced groups and thus reduce the reliance on statistical adjustment [13]. Our adjusted measures of association coincided with our unadjusted estimates for all outcomes (Tables 4–6), demonstrating that matching in the design stage removed confounding by all measured covariates. However, as with all observational studies, matched cohort designs can only ensure balance on observable characteristics [12], and it is possible that residual confounding remained because of unobservable differences between study groups in our study—for example, because of political and socioeconomic considerations by the local authorities in allocating the intervention. Nonetheless, we expect that a large source of unmeasured confounding would be extremely unlikely given the exceptionally good balance between intervention and control groups across a comprehensive set of observable characteristics (Table 2); we believe that these indicators, ascertained by a combination of participant report and direct observation, sufficiently capture all of the main pathways that could confound the relationship between continuous water supply and diarrheal disease.

Furthermore, we observed no differences between study groups in the prevalence of the negative control outcomes (cough/cold and scrapes/bruises) we specified, except for a higher prevalence of scrapes/bruises in the continuous supply group in the wet season. We would expect these symptoms (especially infections leading to coughs and colds) to share a similar set of confounders as reported diarrheal symptoms, such that any spurious relationship due to residual confounding from unmeasured covariates would be expected to manifest itself as an association between continuous water supply and these biologically implausible outcomes. While we did observe an association between scrapes/bruises and continuous supply in the wet season, we cannot think of a confounding mechanism that would lead to spuriously increased prevalence of scrapes/bruises in the continuous supply group only during the wet season, and we interpret this as either a true causal finding or a random chance finding, rather than an indication of unmeasured confounding. Finally, it is also possible that residual confounding would go undetected by our negative control outcomes if there exist confounders of the association between water supply and diarrhea that are not confounders of the association between water supply and cough/cold or scrapes/bruises. The lack of association between continuous water supply and our negative control outcomes in a direction that would suggest confounding nonetheless strengthens the validity of the observed differences in waterborne disease.

#### Measurement bias

Another limitation is that the upgrade to continuous supply was nonblinded by its nature, and our study used caregiver-reported health outcomes. Nonblinded studies with subjectively reported outcomes are vulnerable to reporting bias [42]. However, the outcomes that showed the greatest difference between study groups in our study (blood/mucus in children’s stool and typhoid fever) were of a more severe and specific nature than the more generalized diarrheal symptoms that showed no association with continuous supply (diarrhea and HCGI). We expect that less specific outcomes would be more vulnerable to biased recall; had participants in the continuous supply arm been artificially deflating their reporting of bloody diarrhea and typhoid fever, we would therefore also expect to observe a spurious reduction of a similar or larger magnitude in the overall gastrointestinal symptoms.

Furthermore, the lack of reported differences in the prevalence of the negative control outcomes in the continuous supply group provides an additional robustness check against biased under-reporting of symptoms in recipients of the upgrade [20]. We note that negative control outcomes do not provide definitive evidence for the presence or absence of measurement bias. Negative controls operate on the assumption that the bias structure that affects the study outcomes affects the negative control outcomes in the same way; this might not hold true if study participants are aware that improved water access is expected to reduce diarrheal disease but not coughs/colds and scrapes/bruises (and they therefore artificially deflate their reporting of gastrointestinal symptoms while not deflating their reporting of the negative control outcomes). Nonetheless, the lack of reported reductions in our specified negative control outcomes, taken together with the lack of association between continuous supply and general diarrhea symptoms, supports the conclusion that the associations we observed with bloody diarrhea and typhoid fever are not likely to be explained by biased reporting.

Finally, we note that self-reported incidence of typhoid fever might not accurately represent true cases of typhoid, as the diagnosis in our study setting is typically symptom based and made in local, often informal, clinics. However, we would not expect any misclassification of typhoid fever status to be differential by study arm; any measurement bias introduced by incorrect diagnosis would therefore be expected to bias our findings towards an attenuated association between continuous supply and typhoid fever. Future studies on the association between piped water supply and typhoid fever should ascertain typhoid status with laboratory confirmed diagnosis.

#### Statistical power

We note that our study was powered to detect a 30% relative difference in diarrhea prevalence between study groups. This magnitude of reduction has been observed in trials of household water treatment [43,44]. We assumed that, by removing the need for household storage and thus minimizing point-of-use contamination (in addition to reducing point-of-source contamination in pipelines), continuous supply would be associated with a similar difference. However, continued storage of drinking water eliminated point-of-use water quality benefits, and, notably, the 7% relative difference in diarrhea prevalence that our findings suggest is consistent with the smaller reductions found in studies of point-of-source water quality improvements [43], which our study was not powered to detect. Future studies could address this limitation by focusing on younger (<2 y old) children who are expected to be more susceptible to gastrointestinal illness and have a more sensitive response to changes in water quality [45].

### Generalizability

We would expect our findings to be generalizable to other cities with similar piped water and sanitation infrastructure. Open drains, shallow sewer lines, pit latrines, and septic tanks provide ample sources of fecal contamination in Hubli-Dharwad. The intermittently operated distribution system is characterized by highly degraded pipes, long periods between supply cycles, and areas with persistent low pressure, which provide conditions that enable contamination in pipelines. Many cities in India and other low-income countries share these characteristics. In terms of the implementation of continuous supply, the upgrade in Hubli-Dharwad included complete pipe replacement. Our study therefore cannot discern the impact of continuous water delivery from the impact of replacing leaky pipes. However, leakages in continuously pressurized systems are directed outwards (as opposed to inwards intrusion of exterior contamination in the absence of pressure). We therefore expect that the health benefits from continuous pressure would be maintained to some extent even without replacement of waterlines.

### Scale-up of Continuous Water Supply

Intermittent supply is often considered an inevitable consequence of absolute water scarcity (insufficient water availability) to meet increasing urban demands [46]; however, previous research has shown modest (14.5% to 27.5%) increases in water consumption following a switch from intermittent to continuous supply, and the increase can be as low as 10% in settings where coping mechanisms such as adequate water storage facilities exist [47]. Others have argued that, as long as a minimum threshold number of hours of water service is provided under intermittent supply, allowing users to store the amount of water they need until the following supply cycle, overall water consumption may not change or even decrease under continuous supply by reducing wastage [48]. In settings without absolute water scarcity, management-related scarcity (high rates of leakage in the system, inefficient water use, and poor power supply) and economic scarcity (lack of funds to expand existing intake, treatment, and distribution systems) can be drivers of intermittent supply [46]. Water loss from leaks in pipelines due to poor operation and maintenance can be >40% in low-income countries [49]; potential increases in water use from continuous supply can be accommodated by improved leak management and governance of the supply system. Thus, the factors that must be addressed to upgrade intermittent supplies to continuous supplies are setting specific.

### Conclusions

Continuous, rather than intermittent, water supply was associated with higher drinking water quality and municipal water access but had no significant overall association with gastrointestinal symptoms or weight for age for <5-y-old children in Hubli-Dharwad, India. Storage of drinking water was common despite the upgrade, and point-of-use contamination during storage may have attenuated the health benefits from continuous water supply. Pathogen exposure through nonwaterborne routes such as open sewers, open defecation, and poor domestic and food hygiene might also explain the lack of significant differences in overall diarrheal symptoms and child growth outcomes in this setting.

Continuous supply was associated with significantly lower incidence of typhoid fever in this population and lower prevalence of severe forms of waterborne illness in vulnerable subgroups (dysentery in low-income households). These findings suggest that intermittently operated piped water systems serve as a transmission pathway for waterborne pathogens in this urban population despite centralized treatment. Continuous supply was associated with reduced transmission, especially in the poorer higher-risk segments of the population.

## Supporting Information

S1 Protocol Study protocol. (PDF)

S1 Table STROBE checklist. (DOCX)

S2 Table Comparison of ward characteristics by study group before and after matching. Source of external dataset: Socio-economic survey of Hubli-Dharwad city [18].
(DOCX)

S3 Table Comparison of household characteristics by loss to follow-up status. (DOCX)

S4 Table Water infrastructure and water-related household behaviors by study arm and quarterly round of household visits. (DOCX)

S5 Table Seven-day prevalence of negative control outcomes (children aged <5 y). (DOCX)

S6 Table Seven-day prevalence of child diarrheal illness (children aged <5 y), excluding boundary households. (DOCX)

S7 Table Waterborne disease incidence and child mortality (children aged <2 y) since implementation of continuous supply, excluding boundary households. (DOCX)

S8 Table Inverse probability of censoring-weighting analysis of child diarrheal illness outcomes. (DOCX)

S1 Text Details of statistical methods. (DOCX)

## Abbreviations

BPL: below poverty level
CI: confidence interval
CIR: cumulative incidence ratio
HCGI: highly credible gastrointestinal illness
HH: household
I: incidence
ICC: intraclass correlation coefficient
JMP: Joint Monitoring Programme for Water Supply and Sanitation
KUIDFC: Karnataka Urban Infrastructure Development and Finance Corporation
MDG: Millennium Development Goal
PR: prevalence ratio
SD: standard deviation
STROBE: Strengthening the Reporting of Observational Studies in Epidemiology
UNICEF: United Nations Children's Fund
USD: US dollars
WHO: World Health Organization
