# Supplementary material for: Upgrading a Piped Water Supply from Intermittent to Continuous Delivery and Association with Waterborne Illness: A Matched Cohort Study in Urban India
Source: PLoS Med. 2015 Oct 27;12(10):e1001892. doi: 10.1371/journal.pmed.1001892 (PMC4624240; doi:10.1371/journal.pmed.1001892)
Supplement: S1 Protocol — (PDF) [file pmed.1001892.s001.pdf]

**S1 Protocol. Study protocol**

This study protocol includes additional, related, multidisciplinary studies that were not part of the present epidemiologic core activity. These studies focus on the water quality, quantity and household economics benefits associated with continuous water supply, and their details are described elsewhere [15, 31, 32].

15. Kumpel E, Nelson KL. Comparing Microbial Water Quality in an Intermittent and Continuous Piped Water Supply. Water Res. 2013; Available: http://www.sciencedirect.com/science/article/pii/S0043135413004909

31. Kumpel E. Water Quality and Quantity in Intermittent and Continuous Piped Water Supplies in Hubli-Dharwad, India. Doctoral Dissertation, University of California, Berkeley. 2013.

32. Burt Z, Ray I. Storage and Non-Payment: Persistent Informalities within the Formal Water Supply of Hubli-Dharwad, India. Water Altern. 2014;7: 106–120.

We made the following changes to this protocol after the protocol was filed but before data collection commenced:

1) We added bloody dysentery (blood/mucus in stool), typhoid fever, hepatitis, cholera and <2 child deaths as additional secondary outcomes, in addition to diarrhea (primary outcome) and highly credible gastrointestinal illness as stated in the protocol.

2) We did not conduct measurements of child height and upper arm and head circumference due to logistical constraints. For the same reason, we conducted the weight measurements at the last visit only (as opposed to the first and last visits to the households as stated in the protocol).

3) Instead of propensity score matching, we used a genetic matching algorithm as a superior alternative to match continuous supply wards to intermittent supply wards.

4) Instead of contacting every third house from a selected landmark to assess eligibility for enrollment, we contacted every household as the inclusion criterion of having an <5 child living in the household allowed sufficient geographic spread of enrolled households within the selected study areas.

5) We omitted the inclusion criterion of having lived in the area since the onset of continuous delivery. It was typical for families in our study area to relocate often – limiting the study population to households that remained in the same neighborhood would thus have captured a non-representative population.

6) Instead of excluding households on the immediate continuous-intermittent boundary, we enrolled them and conducted a sensitivity analysis by conducting our data analysis with and without data from these households.

The original protocol was reviewed and approved by the UC Berkeley Institutional Review Board on 06/10/2010, and subsequently amended on 12/08/2010 to make the following changes:

1) The original protocol proposed 12 monthly follow-up visits over one year. We reduced this to 4 visits over one year to avoid respondent fatigue. To maintain the same statistical power with fewer visits, we amended the protocol to enroll a larger number of households.

2) We received additional funding to assess the association between continuous supply and waterborne illness in two separate socioeconomic strata (low vs. high income households), and amended the protocol to double the number of households enrolled to allow statistical power for hypothesis testing within each stratum.

These two changes increased the sample size from 720 households in the original protocol to 4000 households in the amended protocol. We have included the amended protocol (approved on 12/20/2010) here to reflect the study design that was ultimately carried out.

---

**Protocol Title:** Impact Evaluation for Continuous Water Delivery in Hubli-Dharwad, India

**Protocol Status:** APPROVED

**Date Submitted:** 12/08/2010

**Approval Period:** 12/20/2010-06/07/2011

**Important Note:** This Print View may not reflect all comments and contingencies for approval. Please check the comments section of the online protocol.
Questions that appear to not have been answered may not have been required for this submission. Please see the system application for more details.

## * * * Purpose, Background, Collaborative Research * * *

Old CPHS # (for Protocols approved before eProtocol)

**Study Title**

Impact Evaluation for Continuous Water Delivery in Hubli-Dharwad, India

**Complete each section. When a question is not applicable, enter "N/A". Do not leave any sections blank.**

### 1. Purpose

**Provide a brief explanation of the proposed research, including specific study hypothesis, objectives, and rationale.**

OBJECTIVE: The objective of the proposed research is to conduct an impact evaluation for a transition from intermittent water supply, in which water is delivered to consumers a few hours per day or a few days per week on a rotating basis throughout a city, to continuous, fully-pressurized piped water supply. The field work will take place in the city of Hubli-Dharwad, Karnataka, India. As part of a World Bank funded pilot project, 10% of households (70,000 residents) in Hubli-Dharwad were upgraded to continuous delivery in 2007-2008 while the remaining residents continue to receive water once every 4-10 days. We propose to assess the water quality, quantity, health and economic impacts of the upgrade by measuring and comparing these parameters in the continuously and intermittently served areas.

RATIONALE: Intermittent delivery of water is a common form of water supply in developing countries. Intermittent delivery may lead to contamination of water in the pipelines and in the home during storage; it may also limit the quantity of water available, negatively impacting hygiene practices and forcing consumers to rely on contaminated non-municipal sources. Moreover, households must invest in water storage and treatment mechanisms to cope with intermittent delivery. Cumulatively, intermittent supply poses a health threat from increased levels of gastrointestinal illnesses caused by poor water quality and limited water quantity, and creates an economic burden due to health-related expenditures and coping costs. Delivery of continuous, fully-pressurized piped water could be a sustainable and scalable solution to these problems. Proponents of continuous water delivery argue that it will provide safe water and improved health to all; opponents claim that the health impacts may be minimal in the presence of other dominant disease transmission pathways and that the benefits will bypass the poor.

SPECIFIC HYPOTHESES: The proposed research will test two specific hypotheses. (1) Upgrading to continuous water supply will lead to improved water quality, increased quantity of water used in the households, and improved water-related behaviors such as hygiene and sanitation practices. (2) Upgrading to continuous supply will lead to improvements in children's health (specifically longitudinal prevalence of diarrhea and highly credible gastrointestinal illness (HCGI) in children under five, and anthropometric measures including weight, height, upper arm and head circumference) as well as reductions in health-related expenditures and coping costs born by households.

---

**Protocol Title:** Impact Evaluation for Continuous Water Delivery in Hubli-Dharwad, India

**Protocol Status:** APPROVED

**Date Submitted:** 12/08/2010

**Approval Period:** 12/20/2010-06/07/2011

**Important Note:** This Print View may not reflect all comments and contingencies for approval. Please check the comments section of the online protocol. Questions that appear to not have been answered may not have been required for this submission. Please see the system application for more details.

## 2. Background

**Give relevant background (e.g., summarize previous/current related studies) on condition, procedure, product, etc. under investigation, including citations (attach bibliography in Attachments section) if applicable.**

Accessible water, free of contamination from human waste, saves lives and improves productivity and quality of life (Fewtrell et al 2005; Clasen et al 2007). Over half of urban residents in India retrieve water from piped connections (McKenzie and Ray 2009), which meet the UN guidelines of "accessible" and "improved" and are therefore presumed to provide safe drinking water (Coelho et al 2003). However, most piped systems in the developing world operate intermittently, providing water in limited quantities and of questionable quality. Most major Indian cities report water availability to consumers for about four hours per day. Intermittent water delivery exists in an estimated third of urban water supplies in Africa and Latin America, as well as more than half of cities in Asia (WHO/UNICEF 2000).

Intermittent delivery can lead to contamination in pipelines through multiple mechanisms (e.g., intrusion of non-potable water through leaks and cracks, bacterial regrowth) (LeChevallier 2003). Distribution systems in developing countries are especially vulnerable as they are often poorly maintained and intermittent water delivery is common (Lee and Schwab 2005), leading to poor health. There have been no rigorous analyses to our knowledge of the transition from an intermittent to continuous water supply. Although contamination has been shown to occur in intermittent systems (Coelho et al 2003; Tokajian and Hashwa 2003), the exact contamination mechanisms have not been adequately explored, and consistent water quality improvements after conversion to continuous supply have not been documented. Similarly, studies have shown increased risk of gastrointestinal illness after short-term intermittencies in otherwise continuously operated systems (Hunter et al 2005; Nygard et al 2007), but the health implications of having a fully intermittent vs. continuous system have not been documented.

## 3. Collaborative Research

a) **If any non-UCB institutions or individuals are engaged in the research, explain here.**

The Center for Multidisciplinary Development Research (CMDR) in Hubli-Dharwad will be engaged in the research by providing field staff and assisting with data collection and entry. The SDM College of Engineering in Dharwad will assist with water quality testing.

b) **If any non-UCB institutions or individuals are collaborating in the research, complete the table below and attach any relevant IRB approvals in the Attachments section.**

**Non-UCB institutions**

---

**Protocol Title:** Impact Evaluation for Continuous Water Delivery in Hubli-Dharwad, India

**Protocol Status:** APPROVED

**Date Submitted:** 12/08/2010

**Approval Period:** 12/20/2010-06/07/2011

**Important Note:** This Print View may not reflect all comments and contingencies for approval. Please check the comments section of the online protocol. Questions that appear to not have been answered may not have been required for this submission. Please see the system application for more details.

| Institution Name | Individual Contact/ Affiliate of Institution | FWA # | Local IRB Review? (Y or N) | IRB Approval Date | IRB Approval Expiration Date |
|---|---|---|---|---|---|
| Center for Multidisciplinary Development Research (CMDR) | Dr. Nayanatara Nayak | | N | | |

**4. Qualifications of Study Personnel**

a) **Explain expertise of Principal Investigator, Student/Postdoc Researcher, Faculty Sponsor (if applicable), any Co-Investigators or other key personnel listed in the application, and how it relates to their specific roles in the study team.**

Principal Investigator, Jack Colford, is a recognized expert in evaluating health impacts of water and sanitation interventions. He has conducted studies in several developing countries such as India, Bolivia and Guatemala in collaboration with major organizations including the World Health Organization and the World Bank.

Lead student investigator, Ayse Ercumen, has a background in environmental engineering with a focus on water quality, and is now a 3rd year doctoral student in the Division of Epidemiology in the School of Public Health with an emphasis in waterborne infectious disease research. She has extensive understanding of and professional experience in water treatment, contamination, water quality testing and waterborne diseases. Through her class work in the School of Public Health, she has acquired a solid knowledge of epidemiologic study designs, data analysis and the use of statistical software. She has also been working as a graduate student researcher, applying her theoretical skills to help implement a real-world health study under the supervision of UC Berkeley faculty. She will perform the health data collection and analysis.

Co-investigator, Emily Kumpel, is a 3rd year doctoral student with a background in civil and environmental engineering with a focus on water resources. She has experience working with civil society organizations and directly with households in India, and also has experience assisting with survey data collection and water quality testing in India as well as in Madagascar. She will perform the water quality data collection and analysis.

Co-investigator, Zachary Burt, has a background in chemistry and economics, and is now a 3rd year doctoral student in the Energy and Resources Group. He has professional experience in wastewater treatment and pollution control. He has also worked with local communities in a rural part of Morocco on water, hygiene and sanitation projects, learning about effective survey techniques and common practices of water collection, storage and treatment in a low-income context. Through his graduate work in the Energy and Resources Group, he has acquired skills on social science research methods, the political-economy of water issues in a developing country context, risk analysis, cost-benefit analysis and the building of multi-variate statistical models analyzing water demand.

---

| | |
|---|---|
| **Protocol Title:** | Impact Evaluation for Continuous Water Delivery in Hubli-Dharwad, India |
| **Protocol Status:** | APPROVED |
| **Date Submitted:** | 12/08/2010 |
| **Approval Period:** | **12/20/2010-06/07/2011** |
| **Important Note:** | **This Print View may not reflect all comments and contingencies for approval. Please check the comments section of the online protocol. Questions that appear to not have been answered may not have been required for this submission. Please see the system application for more details.** |

**b) ) In case of overseas research, describe the expertise you have, or have access to, which prepares you to conduct research in this location and/or with this subject population, including specific qualifications (e.g., relevant coursework, background, experience, training). Also, explain your knowledge of local community attitudes and cultural norms, and cultural sensitivities necessary to carry out the research. See CPHS Guidelines on Research in an International Setting.**

Ayse Ercumen has lived and worked in two different cities in India (including the proposed research site of Hubli-Dharwad) and in Bangladesh for over 6 months, conducting health research on water-related issues and maintaining close ties to local communities, through which she became closely familiar with South Asian cultural norms and sensitivities. She has basic understanding of written and spoken Hindi but she is not proficient and will be relying on a translator who is fluent in English and Hindi, as well as Kannada (the local language in Karnataka).

Emily Kumpel has lived and worked in two cities in India, including the study site of Hubli-Dharwad as well as Mumbai, for over 10 months. She has worked closely with communities, and established local contacts with students from Hubli-Dharwad and local civil society organizations that are available to assist with cultural issues. She has completed surveys and research related to provision of water and sanitation services in two other countries, Tanzania and Madagascar, and through an internship and continued work with World Health Organization (WHO), completed extensive research and will be lead author on a WHO guide for incorporating cultural sensitivities to the implementation of water supply projects. She has basic understanding of written and spoken Hindi but she is not proficient and will be relying on a translator who is fluent in English and Hindi, as well as Kannada.

Zachary Burt has been the project director for a UC Berkeley based, student-led organization working on water, sanitation and hygiene issues in Hubli-Dharwad for two and a half years. He has spent a substantial amount of time on site, has worked closely with local communities and organizations, and gained close familiarity with the local cultural norms. He has basic understanding of spoken Kannada.

---

---

**Protocol Title:** Impact Evaluation for Continuous Water Delivery in Hubli-Dharwad, India

**Protocol Status:** APPROVED

**Date Submitted:** 12/08/2010

**Approval Period:** 12/20/2010-06/07/2011

**Important Note:** This Print View may not reflect all comments and contingencies for approval. Please check the comments section of the online protocol. Questions that appear to not have been answered may not have been required for this submission. Please see the system application for more details.

## * * * Subject Population * * *

### 5. Subject Population

**a) Describe proposed subject population, stating age range, gender, race, ethnicity, language and literacy.**

The subjects will be selected from among the general population of Hubli-Dharwad, living in the continuous and intermittent water delivery zones. The population of Hubli-Dharwad is a mix of Kannada-speaking Hindus and Hindi/Urdu-speaking Muslims. Participants will be recruited from both ethnic/religious groups. According to the 2001 census, the general literacy rate is 0.71 and 0.73 in Hubli and Dharwad, respectively, and the female literacy rate is 0.65 in Hubli and 0.68 in Dharwad. Both literate and illiterate subjects will be recruited into the study. Participants will be households with children under the age of five.

**b) State total number of subjects planned for the study and how many must be recruited to obtain this sample size. Explain how number of subjects needed to answer the research question was determined.**

2000 households will be recruited into each study arm (intermittent vs. continuous water delivery), with a total of 4000 households. With one adult participants in each enrolled household and assuming an average of two children under the age of five per household, this will result in 12,000 participants. If there are more than two children under five in a household, all of them will be recruited into the study. Each house will be visited once every three months for the duration of a year. This will yield 80% power to detect our hypothesized health impact in two different income strata, using a one-sided alpha of 5% (assuming 10% loss to follow-up and adjusting for clustering).

An additional 700 households will be contacted solely for water quality testing, and will not participate in the full health study. This number was determined based on the availability of water quality testing equipment to the investigators. In these households, the household head will be recruited to provide a water sample from their tap and storage containers to the investigators. In addition to providing water quality samples, these participants will be asked to answer questions from a brief sanitary survey about their water collection practices.

There will be a total of 12,700 participants (4000 households with one adult and an average of two children per household for the health study, and 700 additional individuals for water quality testing).

**c) If any proposed subjects are children/minors, prisoners, pregnant women, those with physical or cognitive impairments, or others who are considered vulnerable to coercion or undue influence, state rationale for their involvement.**

Data on the occurrence of diarrhea and highly credible gastrointestinal illness (HCGI) will be collected on children under the age of five as this age group is particularly vulnerable to and suffers high morbidity and mortality from those illnesses, with almost 2 million annual deaths, 85% of them in young children in developing countries. Anthropmetric measures will also be taken on the children as persistent diarrheal illness can lead to stunted growth. The proposed evaluation of continuous water delivery will fill an important information gap on whether this intervention leads to improvements in child health and the findings can be applied to develop water-related interventions that will effectively reduce childhood

mortality from diarrheal illnesses across the developing world.

The information on the occurrence of diarrhea and HCGI will be obtained from the primary caretaker of the children, and not the children themselves. Anthropometric measurements (weigth, height, head and upper arm circumference) will be taken on the children.

## 6. Recruitment

a) **Explain how, where, when, and by whom prospective subjects will be identified/selected and approached for study participation. If researcher is subject's instructor, physician, or job supervisor, or if vulnerable subject groups will be recruited, explain what precautions will be taken to minimize potential coercion or undue influence to participate. See CPHS Guidelines on Recruitment for more information.**

The pilot upgrade to continuous delivery has already been implemented in 2007-2008. Hubli-Dharwad is divided into 67 sub-units called wards. Eight wards were selected to receive continuous supply based on the ability to hydraulically isolate the distribution network serving the ward, as well as ward size and socioeconomic status (SES).

We will compare water quality, health and coping costs in households in intermittently supplied zones versus continuously supplied zones to assess the impact of the upgrade to continuous delivery. However, since the zones to receive continuous supply were selected non-randomly by the municipality, a direct comparison between continuous and intermittent supply wards would be confounded by neighborhood-related covariates. We will therefore use a matched design to help control confounding by substantially reducing imbalances in potential confounders between continuous and intermittent supply wards. We will use propensity score matching to allow matching on a large set of covariates, and eight intermittent delivery zones will be selected based on their covariate patterns as controls for the eight wards in which continuous delivery has been implemented. The propensity score matching will be conducted in summer 2010.

After a suitable set of control wards has been selected using propensity score matching, an SES-stratified random subset of households will be enrolled into the study in each selected ward in fall 2010. The field staff will be asked to select a random starting point in the ward and contact every third house until the desired number of households in each SES stratum has been recruited in each ward. For each household in the continuous supply wards, one household will be enrolled in the intermittent supply wards. The 700 additional households selected solely for water quality testing will be selected by Emily Kumpel based on existing hydraulic maps of the city's water distribution system.

In order to avoid coercion and undue influence on economically disadvantaged subjects, there will be no benefits offered in exchange for participation. To ensure fully informed consent by educationally disadvantaged participants and minors, the language of the consent script will be simple and non-technical, and appropriate for the level of understanding of these vulnerable subjects.

b) **Describe any recruitment materials (e.g., letters, flyers, advertisements [note type of media/where posted], scripts for verbal recruitment, etc.) and letter of permission/cooperation from institutions, agencies or organizations where off-site subject recruitment will take place (e.g., another UC campus, clinic, school district). Attach these documents in Attachments section.**

---

**Protocol Title:** Impact Evaluation for Continuous Water Delivery in Hubli-Dharwad, India

**Protocol Status:** APPROVED

**Date Submitted:** 12/08/2010

**Approval Period:** 12/20/2010-06/07/2011

**Important Note:** **This Print View may not reflect all comments and contingencies for approval. Please check the comments section of the online protocol. Questions that appear to not have been answered may not have been required for this submission. Please see the system application for more details.**

Recruitment of participants will occur verbally. The investigators' translator will introduce herself and investigators, and ask the subject if the household meets the inclusion criteria. If the inclusion criteria are met, the translator will recite a verbal recruitment script giving information on the content and length of the study, including the number of follow-up visits. If the subjects express interest in participating, the translator will proceed with the verbal informed consent process.

As recruitment and informed consent will occur simultaneously at the first contact with the household, the recruitment script has been combined with the consent script and attached in the informed consent section.

## 7. Screening

**a) Provide criteria for subject inclusion and exclusion. If any inclusion/exclusion criteria are based on gender, race, or ethnicity, explain rationale for restrictions.**

Two inclusion criteria will be applied during the recruitment process: (1) having at least one child under age five living in the household, (2) having lived in the area since the onset of continuous delivery. To prevent spillover effects, which may occur due to water sharing and the infectious nature of diarrhea, households located in the immediate vicinity of the dividing line between the continuous and intermittent supply zones will be excluded from recruitment.

**b) If prospective subjects will be screened via tests, interviews, etc., prior to entry into the "main" study, explain how, where, when, and by whom screening will be done. NOTE: Consent must be obtained for screening procedures as well as "main" study procedures. As appropriate, either: 1) create a separate "Screening Consent Form;" or 2) include screening information within the consent form for the main study.**

At the first contact, the investigators will ask the subjects whether they are the parent or primary caretaker of at least one child under the age of five and have lived in the study community since the onset of continuous water delivery. Households not meeting these criteria will not be recruited into the study. If the household meets the criteria, the translator will proceed to recruitment followed by informed consent.

## 8. Compensation and Costs

a)

Describe plan for compensation of subjects. If no compensation will be provided, this should be stated. If subjects will be compensated for their participation, explain in detail about the amount and methods/ terms of payment.

- Include any provisions for partial payment if subject withdraws before study is complete.

- When subjects are required to provide Social Security Number in order to be paid, this data must be

---



collected separately from consent documentation. If applicable, describe security measures that will be used to protect subject confidentiality.

**- If non-monetary compensation (e.g., course credit, services) will be offered, explain how it will be provided.**

There will be no monetary compensation for participation in the study. The participants will be given small prizes such as soap, safe water storage containers and chlorine tablets as a token of appreciation at the last follow-up visit. Moreover, small gifts for children such as crayons, notebooks, pens and pencils will be given out during each visit.

b) **Discuss reasoning behind amount/method/terms of compensation, including appropriateness of compensation for the study population and avoiding undue influence to participate.**

Compensation was kept to a minimum to avoid unduly influencing potential subjects to participate. Prizes were selected to continue to improve health outcomes in the households after the study and encourage purchase of items like soap and chlorine in the future.

c) **Costs to Subjects. If applicable, describe any costs/charges which subjects or their insurance carriers will be expected to pay. (If there are no costs to subjects or their insurers, this should be stated.)**

There will be no costs to the subjects.

---

---

| | |
|---|---|
| **Protocol Title:** | Impact Evaluation for Continuous Water Delivery in Hubli-Dharwad, India |
| **Protocol Status:** | APPROVED |
| **Date Submitted:** | 12/08/2010 |
| **Approval Period:** | **12/20/2010-06/07/2011** |
| **Important Note:** | **This Print View may not reflect all comments and contingencies for approval. Please check the comments section of the online protocol. Questions that appear to not have been answered may not have been required for this submission. Please see the system application for more details.** |

## * * * Study Procedures, Alternatives to Participation * * *

### 9. Study Procedures

a) **Describe in chronological order of events how the research will be conducted, providing information about all study procedures (e.g., all interventions/interactions with subjects, data collection procedures etc.), including follow-up procedures.**

Upon recruitment and informed consent, the investigators will administer a questionnaire to the primary caretaker in the household to collect data on children's health, health-related expenditures and coping costs, water quantity used, and water-related behaviors such as water storage, hygiene and sanitation practices. Information on basic demographics will also be collected in this initial questionnaire to allow for control of potential confounders in the data analysis phase. After the initial interview, the households will be visited three more times, and a questionnaire will be administered to collect the same data as in the initial questionnaire except for the demographic information. Water samples will be collected from the household's tap and stored water during every other visit. Anthropometric measurements will be taken on the children under the age of five during the first and last visits to each household. The additional 700 households selected solely for water quality sampling will only be visited once.

b) **Explain who will conduct the procedures, where and when they will take place. Indicate frequency and duration of visits/sessions, as well as total time commitment for the study.**

A local team of experienced field workers, trained in health data collection and water quality sampling by the investigators, will conduct the procedures. The household visits will occur once every three months for the duration of a year (fall 2010-fall 2011), and are expected to take less than an hour to complete, with the exception of the first and last visits which involve anthropometric measurements. The additional 700 households selected solely for water quality sampling will also only be visited once.

c) **Identify any procedures that are experimental/ investigational and explain how they differ from standard procedures (medical, psychological, educational). If applicable, distinguish between procedures that the subject would undergo regardless of enrollment in the study and procedure done specifically for study.**

There are no experimental/investigatinal procedures involved in the study.

d) **If a placebo will be used, provide rationale and explain why active control is not appropriate.**

No placebo will be used in the study.

e) **If any type of deception or lack of full disclosure will be used, explain what it will entail, why it is justified, and what the plans are to debrief subjects. See CPHS Guidelines on Deception and Incomplete Disclosure for more information. Any debreifing materials should be included in the Attachments section.**

There will be no deception or lack of full disclosure involved in the study.

---

**Protocol Title:** Impact Evaluation for Continuous Water Delivery in Hubli-Dharwad, India

**Protocol Status:** APPROVED

**Date Submitted:** 12/08/2010

**Approval Period:** 12/20/2010-06/07/2011

**Important Note:** This Print View may not reflect all comments and contingencies for approval. Please check the comments section of the online protocol.
Questions that appear to not have been answered may not have been required for this submission. Please see the system application for more details.

f) **State if audio or video taping will occur. Describe what will become of the tapes after the project (e.g., shown at scientific meetings, erased) and final disposition of the tapes.**

No audio or video taping will occur.

## 10. Alternatives to Participation

**Describe appropriate alternative resources, procedures, courses of treatment, if any, that are available to prospective subjects. If there are no appropriate alternatives to study participation, this should be stated. If the study does not involve treatment/intervention, enter "N/A" here.**

N/A

---
