# Supplementary material for: Upgrading a Piped Water Supply from Intermittent to Continuous Delivery and Association with Waterborne Illness: A Matched Cohort Study in Urban India
Source: PLoS Med. 2015 Oct 27;12(10):e1001892. doi: 10.1371/journal.pmed.1001892 (PMC4624240; doi:10.1371/journal.pmed.1001892)
Supplement: S1 Table — (DOCX) [file pmed.1001892.s002.docx]

**S1 Table. STROBE checklist**

| **ITEM** | **DESCRIPTION** | **REPORTED IN SECTION** |
| --- | --- | --- |
| **Title and Abstract** | | |
| 1a | Indicate the study’s design with a commonly used term in the title or the abstract | Title, Abstract |
| 1b | Provide in the abstract an informative and balanced summary of what was done and what was found | Abstract |
| **Introduction** | | |
| Background/rationale | | |
| 2 | Explain the scientific background and rationale for the investigation being reported | Introduction |
| Objectives | | |
| 3 | State specific objectives, including any prespecified hypotheses | Introduction, Methods (Statistical Methods – Statistical Analysis) |
| **Methods** | | |
| Study Design | | |
| 4 | Present key elements of study design early in the paper | Methods (Selection of Study Areas; Participant Selection and Enrollment; Outcome Definition and Measurement) |
| Setting | | |
| 5 | Describe the setting, locations, and relevant dates, including periods of recruitment, exposure, follow-up, and data collection | Methods (Study Setting; Selection of Study Areas, Participant Selection and Enrollment, Outcome Definition and Measurement) |
| Participants | | |
| 6a | Give the eligibility criteria, and the sources and methods of selection of participants. Describe methods of follow-up | Methods (Participant Selection and Enrollment) |
| 6b | For matched studies, give matching criteria and number of exposed and unexposed. | Methods (Study Setting; Selection of Study Areas, Participant Selection and Enrollment) |
| Variables | | |
| 7 | Clearly define all outcomes, exposures, predictors, potential confounders, and effect modifiers. Give diagnostic criteria, if applicable | Methods (Outcome Definition and Measurement) |
| Data Sources and Management | | |
| 8 | For each variable of interest, give sources of data and details of methods of assessment (measurement). Describe comparability of assessment methods if there is more than one group | Methods (Outcome Definition and Measurement) |
| Bias | | |
| 9 | Describe any efforts to address potential sources of bias | Methods (Outcome Definition and Measurement; Statistical Methods – Statistical Analysis) |
| Study Size | | |
| 10 | Explain how the study size was arrived at | Methods (Statistical Methods – Sample Size) |
| Quantitative Variables | | |
| 11 | Explain how quantitative variables were handled in the analyses. If applicable, describe which groupings were chosen and why | Methods (Statistical Methods – Statistical Analysis) |
| Statistical Methods | | |
| 12a | Describe all statistical methods, including those used to control for confounding | Methods (Statistical Methods – Statistical Analysis) |
| 12b | Describe any methods used to examine subgroups and interactions | Methods (Statistical Methods – Statistical Analysis) |
| 12c | Explain how missing data were addressed | Methods (Statistical Methods – Statistical Analysis) |
| 12d | If applicable, explain how loss to follow-up was addressed | Methods (Statistical Methods – Statistical Analysis) |
| 12e | Describe any sensitivity analyses | N/A |
| **Results** | | |
| Participants | | |
| 13a | Report numbers of individuals at each stage of study—eg numbers potentially eligible, examined for eligibility, confirmed eligible, included in the study, completing follow-up, and analysed | Results (Characteristics of Study Participants; Figure 3) |
| 13b | Give reasons for non-participation at each stage | Results (Figure 3) |
| 13c | Consider use of a flow diagram | Results (Figure 3) |
| Descriptive data | | |
| 14a | Give characteristics of study participants (eg demographic, clinical, social) and information on exposures and potential confounders | Results (Characteristics of Study Wards; Characteristics of Study Participants; Table 2) |
| 14b | Indicate number of participants with missing data for each variable of interest | Results (Tables 4-6) |
| 14c | Summarise follow-up time (eg, average and total amount) | Results (Figure 3) |
| Outcome Data | | |
| 15 | Report numbers of outcome events or summary measures over time | Results (Waterborne Diseases and Weight-for-Age; Tables 4-6) |
| Main Results | | |
| 16a | Give unadjusted estimates and, if applicable, confounder-adjusted estimates and their precision (eg, 95% confidence interval). Make clear which confounders were adjusted for and why they were included | Results (Waterborne Diseases and Weight-for-Age; Tables 4-6) |
| 16b | Report category boundaries when continuous variables were categorized | N/A |
| 16c | If relevant, consider translating estimates of relative risk into absolute risk for a meaningful time period | N/A |
| Other Analyses | | |
| 17 | Report other analyses done—eg analyses of subgroups and interactions, and sensitivity analyses | Results (Waterborne Diseases and Weight-for-Age; Tables 4-5) |
| **Discussion** | | |
| Key Results | | |
| 18 | Summarise key results with reference to study objectives | Discussion (Continuous Supply and Gastrointestinal Illness) |
| Limitations | | |
| 19 | Discuss limitations of the study, taking into account sources of potential bias or imprecision. Discuss both direction and magnitude of any potential bias | Discussion (Limitations) |
| Interpretation | | |
| 20 | Give a cautious overall interpretation of results considering objectives, limitations, multiplicity of analyses, results from similar studies, and other relevant evidence | Discussion |
| Generalisability | | |
| 21 | Discuss the generalisability (external validity) of the study results | Discussion (Generalizability) |
| **Other Information** | | |
| Funding | | |
| 22 | Give the source of funding and the role of the funders for the present study and, if applicable, for the original study on which the present article is based | Funding |
