# Supplementary material for: Upgrading a Piped Water Supply from Intermittent to Continuous Delivery and Association with Waterborne Illness: A Matched Cohort Study in Urban India
Source: PLoS Med. 2015 Oct 27;12(10):e1001892. doi: 10.1371/journal.pmed.1001892 (PMC4624240; doi:10.1371/journal.pmed.1001892)
Supplement: S2 Table — Source of external dataset: Socio-economic survey of Hubli-Dharwad city [18]. (DOCX) [file pmed.1001892.s003.docx]

**S2 Table. Comparison of ward characteristics by study group before and after matching. Source of external dataset: Socio-economic survey of Hubli-Dharwad city [18].**

|  | Full Set of Intermittent | |  | Matched Set of Intermittent | |  | Continuous Supply |
| --- | --- | --- | --- | --- | --- | --- | --- |
|  | Supply Wards (N=59) | |  | Supply Wards (N=8) | |  | Wards (N=8) |
|  | Mean/% | Standardized Difference ^a^ |  | Mean/% | Standardized Difference ^a^ |  | Mean/% |
| **Demographics and socioeconomics** |  |  |  |  |  |  |  |
| Mean number of persons per household | 5.1 | -68 |  | 5.0 | -30 |  | 4.8 |
| Mean number of children aged <5 y per household | 1.4 | -115 |  | 1.4 | -85 |  | 1.3 |
| % of children aged < 5 y | 7.7 | -60 |  | 6.9 | -14 |  | 6.7 |
| % of males | 51.8 | 17 |  | 52.5 | -26 |  | 52.0 |
| % of married individuals | 47.4 | 100 |  | 48.6 | 57 |  | 50.2 |
| % of illiterate females | 17.7 | -13 |  | 16.4 | 15 |  | 17.1 |
| % of individuals working as agricultural laborer | 12.0 | 6 |  | 12.3 | 2 |  | 12.4 |
| % of non-Hindu households | 26.0 | -83 |  | 22.2 | -40 |  | 18.7 |
| % of scheduled caste or tribe households | 14.6 | -94 |  | 10.4 | 4 |  | 10.5 |
| % of slum households (self-report) | 22.8 | 10 |  | 23.7 | 6 |  | 25.0 |
| % of migrant households | 14.5 | -63 |  | 13.1 | -47 |  | 8.6 |
| % of BPL card holder households ^b^ | 25.9 | -8 |  | 26.7 | -14 |  | 24.8 |
| % of households with income <US$350/year | 12.2 | -47 |  | 11.6 | -37 |  | 9.5 |
| % of households that borrow money | 12.6 | -117 |  | 11.4 | -94 |  | 6.2 |
| % of households that save money | 6.9 | -16 |  | 7.9 | -30 |  | 5.8 |
| % of households that report having sufficient income | 85.1 | 177 |  | 89.6 | 103 |  | 95.8 |
| % of households that own agricultural land | 8.0 | -75 |  | 7.0 | -55 |  | 4.1 |
| % of households that own their home | 65.9 | 92 |  | 72.5 | 16 |  | 73.9 |
| % of *pukka* homes ^c^ | 71.5 | 15 |  | 75.6 | -11 |  | 73.9 |
| % of one-room homes | 6.3 | -213 |  | 5.2 | -141 |  | 2.9 |
| % of households that have: |  |  |  |  |  |  |  |
| Electricity | 94.4 | 49 |  | 95.4 | 11 |  | 95.8 |
| TV | 77.6 | 2 |  | 78.5 | -3 |  | 78.0 |
| Phone | 38.4 | 1 |  | 40.4 | -8 |  | 38.7 |
| Fridge | 16.0 | -10 |  | 17.0 | -17 |  | 14.6 |
| Grinder | 53.7 | 13 |  | 55.5 | 1 |  | 55.6 |
| Radio | 39.4 | 51 |  | 42.5 | 30 |  | 46.7 |
| LPG cylinder | 66.7 | 32 |  | 70.8 | 7 |  | 71.9 |
| Bicycle | 26.9 | 41 |  | 30.4 | 15 |  | 32.4 |
| Motorcycle | 29.7 | 7 |  | 35.6 | -33 |  | 30.6 |
| Car | 3.5 | -6 |  | 4.6 | -39 |  | 3.3 |
| Computer | 3.1 | -8 |  | 3.2 | -11 |  | 2.9 |
| Agricultural assets | 2.3 | -119 |  | 1.7 | -72 |  | 0.8 |
| Livestock | 1.9 | -1 |  | 1.3 | 17 |  | 1.9 |
| Number of households in ward | 2,313.6 | -39 |  | 2,433.0 | -67 |  | 2,148.9 |
| Ward infrastructure index ^d^ | 0.2 | -66 |  | 0.2 | -69 |  | 0.1 |
| **Water, sanitation, and hygiene conditions** |  |  |  |  |  |  |  |
| % of households with own tap | 79.5 | 136 |  | 88.9 | 25 |  | 91.1 |
| % of households receiving water every 5 or more d | 7.7 | -313 |  | 2.8 | -72 |  | 1.3 |
| % of households paying >US$2/month for water | 3.5 | -51 |  | 4.1 | -77 |  | 2.4 |
| % of households with own latrine | 74.5 | 32 |  | 79.1 | 5 |  | 80.0 |
| % of households served by open drain | 8.4 | 65 |  | 12.0 | 55 |  | 31.3 |
| % of households with designated garbage bin or collection at door | 47.1 | 9 |  | 45.6 | 16 |  | 49.0 |
| % of households with garbage cleared regularly by municipality | 37.5 | -9 |  | 37.9 | -11 |  | 35.5 |
| % of households with health expenditures >US$2/month | 24.5 | 46 |  | 21.1 | 64 |  | 33.3 |
| % of households receiving healthcare at private hospital or clinic | 68.1 | -70 |  | 69.0 | -74 |  | 53.5 |

Abbreviations: BPL, below poverty level; LPG, liquid propane gas.

^a^ Standardized difference is the difference between ward-level means in two study arms divided by the ward-level standard deviation in continuous supply arm. ^b^ A BPL card is issued by the government based on household income.

^c^ *Pukka* refers to concrete or reinforced cement concrete. ^d^ Ward infrastructure index is a combined metric based on the number of schools, hospitals and other amenities in the ward.
