# Supplementary material for: Upgrading a Piped Water Supply from Intermittent to Continuous Delivery and Association with Waterborne Illness: A Matched Cohort Study in Urban India
Source: PLoS Med. 2015 Oct 27;12(10):e1001892. doi: 10.1371/journal.pmed.1001892 (PMC4624240; doi:10.1371/journal.pmed.1001892)
Supplement: S3 Table — (DOCX) [file pmed.1001892.s004.docx]

**S3 Table. Comparison of household characteristics by loss to follow-up status**

|  | HHs that completed | |  | HHs lost to | |
| --- | --- | --- | --- | --- | --- |
|  | study (N=3305) | |  | follow-up (N=617) | |
|  | N | Mean/% |  | N | Mean/% |
| **Demographics and socioeconomics** |  |  |  |  |  |
| Mean number of persons per household | 3302 | 6.7 |  | 617 | 5.4 |
| Mean number of children aged <5 y per household | 3302 | 1.4 |  | 617 | 1.4 |
| Mean age of primary caregiver of children aged <5 y | 3285 | 27.1 |  | 616 | 26.5 |
| Mean monthly household income (USD) | 2554 | 202.2 |  | 474 | 183.4 |
| Mean number of rooms in household | 3301 | 2.4 |  | 617 | 2.0 |
| % of households with: |  |  |  |  |  |
| *Pukka* roof ^a^ | 3301 | 44.9 |  | 617 | 43.1 |
| *Pukka* walls ^a^ | 3240 | 59.8 |  | 358 | 60.3 |
| *Pukka* floor ^a^ | 3240 | 95.8 |  | 358 | 96.1 |
| Fridge | 3305 | 28.1 |  | 617 | 26.1 |
| Motorcycle | 3305 | 49.3 |  | 617 | 41.7 |
| Mobile phone | 3305 | 90.4 |  | 617 | 88.7 |
| % of households owning at least one home | 3300 | 70.7 |  | 617 | 38.7 |
| % of self-employed father | 3290 | 34.7 |  | 617 | 30.1 |
| % of illiterate mother | 3294 | 9.2 |  | 616 | 9.9 |
| % Hindu | 3301 | 69.7 |  | 617 | 69.0 |
| **Water, sanitation, and hygiene indicators** |  |  |  |  |  |
| % of households with handwashing facility: |  |  |  |  |  |
| Inside the household | 3302 | 73.7 |  | 617 | 72.9 |
| In yard | 3302 | 25.1 |  | 617 | 24.1 |
| No specific place | 3302 | 1.2 |  | 617 | 2.9 |
| % of households with sanitation access: |  |  |  |  |  |
| Private latrine | 3241 | 91.4 |  | 359 | 91.9 |
| Public latrine | 3241 | 4.9 |  | 359 | 3.9 |
| No latrine | 3241 | 3.7 |  | 359 | 4.2 |
| % of households where children aged <5 y defecate: |  |  |  |  |  |
| In latrine or potty | 3236 | 65.4 |  | 359 | 61.3 |
| In area within household compound | 3236 | 18.0 |  | 359 | 18.1 |
| In area outside household compound | 3236 | 17.9 |  | 359 | 21.2 |
| % of households with sewerage in vicinity: |  |  |  |  |  |
| Underground piped sewer | 3235 | 76.3 |  | 358 | 77.1 |
| Open drain or open sewage canal | 3235 | 73.6 |  | 358 | 70.9 |
| % of households with garbage disposal: |  |  |  |  |  |
| In open heap | 3240 | 29.8 |  | 358 | 33.8 |
| Designed bin | 3240 | 45.3 |  | 358 | 41.6 |
| Collected at the door | 3240 | 21.6 |  | 358 | 21.2 |

Abbreviations: HH, household; USD, US dollars.

^a^ Pukka refers to concrete or reinforced cement concrete.
