# Supplementary material for: Upgrading a Piped Water Supply from Intermittent to Continuous Delivery and Association with Waterborne Illness: A Matched Cohort Study in Urban India
Source: PLoS Med. 2015 Oct 27;12(10):e1001892. doi: 10.1371/journal.pmed.1001892 (PMC4624240; doi:10.1371/journal.pmed.1001892)
Supplement: S4 Table — (DOCX) [file pmed.1001892.s005.docx]

**S4 Table. Water infrastructure and water-related household behaviors by study arm and quarterly round of household visits**

|  | Continuous Supply (%) | | | |  | Intermittent Supply (%) | | | |
| --- | --- | --- | --- | --- | --- | --- | --- | --- | --- |
|  | Round 1 | Round 2 | Round 3 | Round 4 |  | Round 1 | Round 2 | Round 3 | Round 4 |
|  | Nov-Mar | Mar-Jul | Jul-Nov | Nov-Feb |  | Nov-Mar | Mar-Jul | Jul-Nov | Nov-Feb |
| **Water infrastructure and services** |  |  |  |  |  |  |  |  |  |
| Customer satisfaction: |  |  |  |  |  |  |  |  |  |
| Tap water does not smell or look dirty | 69 | 71 | 66 | 84 |  | 60 | 65 | 46 | 68 |
| Happy with tap water quality | 74 | 81 | 66 | 89 |  | 45 | 52 | 48 | 76 |
| Happy with tap water quantity | 93 | 97 | 86 | 99 |  | 57 | 59 | 62 | 96 |
| Happy with tap water pressure | 94 | 92 | 80 | 94 |  | 41 | 44 | 50 | 83 |
| **Water-related household behaviors** |  |  |  |  |  |  |  |  |  |
| Retrieves drinking water from: |  |  |  |  |  |  |  |  |  |
| Tap connected directly to waterline | 10 | 12 | 8 | 8 |  | 4 | 2 | 1 | 0 |
| Tap connected to overhead tank | 2 | 1 | 0 | 0 |  | 0 | 0 | 0 | 0 |
| Storage container | 79 | 76 | 75 | 76 |  | 85 | 83 | 81 | 83 |
| Commercial water treatment device | 8 | 9 | 15 | 15 |  | 10 | 12 | 16 | 16 |
| Bottled water | 1 | 2 | 2 | 1 |  | 1 | 2 | 2 | 1 |
| Collects water from other sources: |  |  |  |  |  |  |  |  |  |
| Borewell (public or private) | 3 | 8 | 6 | 5 |  | 38 | 51 | 43 | 17 |
| Water truck | 0 | 0 | 0 | 0 |  | 3 | 3 | 1 | 0 |
