# Supplementary material for: Upgrading a Piped Water Supply from Intermittent to Continuous Delivery and Association with Waterborne Illness: A Matched Cohort Study in Urban India
Source: PLoS Med. 2015 Oct 27;12(10):e1001892. doi: 10.1371/journal.pmed.1001892 (PMC4624240; doi:10.1371/journal.pmed.1001892)
Supplement: S5 Table — (DOCX) [file pmed.1001892.s006.docx]

**S5 Table. Seven-day prevalence of negative control outcomes (children aged <5 y)**

|  | Intermittent Supply | |  | Continuous Supply | | | | | | | |
| --- | --- | --- | --- | --- | --- | --- | --- | --- | --- | --- | --- |
|  | N | Prevalence % |  | N | Prevalence % | PR | 95% CI ^a^ | Adjusted PR ^b^ | 95% CI ^a^ | Regression p-value | Permutation test p-value ^c^ |
| Main analysis |  |  |  |  |  |  |  |  |  |  |  |
| Cough or cold | 10021 | 41.5 |  | 10052 | 41.1 | 0.99 | (0.95–1.03) | 1.00 | (0.96–1.05) | 0.96 | 1.00 |
| Scrapes or bruises | 10017 | 5.0 |  | 10048 | 5.6 | 1.12 | (0.99–1.29) | 1.12 | (0.97–1.29) | 0.10 | 0.51 |
| Subgroup analysis by wealth |  |  |  |  |  |  |  |  |  | Interaction p-value ^d^ |  |
| **Above median wealth** |  |  |  |  |  |  |  |  |  |  |  |
| Cough or cold ^e^ | 5043 | 39.4 |  | 5038 | 38.4 | 0.98 | (0.92–1.04) | 0.98 | (0.92–1.05) | 0.47 |  |
| Scrapes or bruises | 5041 | 4.4 |  | 5036 | 5.1 | 1.15 | (0.94–1.42) | 1.13 | (0.91–1.38) | 0.95 |  |
| **Below median wealth** |  |  |  |  |  |  |  |  |  |  |  |
| Cough or cold ^e^ | 4972 | 43.5 |  | 4988 | 43.7 | 1.00 | (0.95–1.06) | 1.02 | (0.95–1.08) | -- |  |
| Scrapes or bruises | 4970 | 5.5 |  | 4986 | 6.1 | 1.10 | (0.92–1.31) | 1.12 | (0.92–1.35) | -- |  |
| Subgroup analysis by rainfall |  |  |  |  |  |  |  |  |  | Interaction p-value ^d^ |  |
| **Dry period (>10 d after rain)** | |  |  |  |  |  |  |  |  |  |  |
| Cough or cold ^e^ | 4284 | 44.5 |  | 4342 | 44.5 | 1.00 | (0.95–1.05) | 1.02 | (0.96–1.08) | 0.43 |  |
| Scrapes or bruises | 4282 | 4.7 |  | 4342 | 4.5 | 0.97 | (0.79–1.19) | 0.96 | (0.77–1.18) | 0.05 |  |
| **Wet period (<10 d after rain)** | |  |  |  |  |  |  |  |  |  |  |
| Cough or cold ^e^ | 5737 | 39.2 |  | 5710 | 38.5 | 0.98 | (0.93–1.04) | 0.99 | (0.93–1.05) | -- |  |
| Scrapes or bruises | 5735 | 5.1 |  | 5706 | 6.3 | 1.23 | (1.05–1.45) | 1.23 | (1.04–1.47) | -- |  |

Abbreviations: PR, prevalence ratio; CI, confidence interval.

^a^ CIs obtained by bootstrapping within strata of wards with clustering at household level.

^b^ Adjusted for child age, child sex, season, household socioeconomic status, religion, handwashing infrastructure, latrine ownership, sewerage, and garbage disposal; we only included covariates in the adjusted models that could not plausibly be impacted by the continuous supply intervention. ^c^ p-value from Wilcoxon rank-sum permutation test; the permutation test is conservative relative to the CIs around the PR because it tests the null hypothesis that the two groups have the same distribution as opposed to the null hypothesis of no effect on average. ^d^ p-value for interaction from generalized linear model with interaction terms. ^e^ Adjusted PR obtained with Poisson regression due to non-convergence of log-binomial regression.
