# Supplementary material for: Upgrading a Piped Water Supply from Intermittent to Continuous Delivery and Association with Waterborne Illness: A Matched Cohort Study in Urban India
Source: PLoS Med. 2015 Oct 27;12(10):e1001892. doi: 10.1371/journal.pmed.1001892 (PMC4624240; doi:10.1371/journal.pmed.1001892)
Supplement: S6 Table — (DOCX) [file pmed.1001892.s007.docx]

**S6 Table. Seven-day prevalence of child diarrheal illness (children aged <5 y), excluding boundary households**

|  | Intermittent Supply | |  | Continuous Supply | | | | | | |
| --- | --- | --- | --- | --- | --- | --- | --- | --- | --- | --- |
|  | N | Prevalence % |  | N | Prevalence % | PR | 95% CI ^a^ | Adjusted PR ^b^ | 95% CI ^a^ | p-value ^c^ |
| HCGI | 10000 | 11.3 |  | 7664 | 11.8 | 1.04 | (0.94–1.14) | 1.03 | (0.93–1.14) | 0.67 |
| Diarrhea (primary outcome) | 10019 | 8.4 |  | 7679 | 7.9 | 0.94 | (0.83–1.06) | 0.93 | (0.82–1.05) | 0.96 |
| Blood or mucus in stool | 10016 | 1.9 |  | 7677 | 1.6 | 0.84 | (0.65–1.06) | 0.83 | (0.62–1.10) | 0.35 |

Abbreviations: PR, prevalence ratio; CI, confidence interval; HCGI, highly credible gastrointestinal illness.

^a^ CIs obtained by bootstrapping within strata of wards with clustering at household level. ^b^ Adjusted for child age, child sex, season, household socioeconomic status, religion, handwashing infrastructure, latrine ownership, sewerage, and garbage disposal; we only included covariates in the adjusted models that could not plausibly be impacted by the continuous supply intervention. ^c^ p-value from Wilcoxon rank-sum permutation test; the permutation test is conservative relative to the CIs around the PR because it tests the null hypothesis that the two groups have the same distribution as opposed to the null hypothesis of no effect on average.
