# Supplementary material for: Upgrading a Piped Water Supply from Intermittent to Continuous Delivery and Association with Waterborne Illness: A Matched Cohort Study in Urban India
Source: PLoS Med. 2015 Oct 27;12(10):e1001892. doi: 10.1371/journal.pmed.1001892 (PMC4624240; doi:10.1371/journal.pmed.1001892)
Supplement: S7 Table — (DOCX) [file pmed.1001892.s008.docx]

S7 Table. Waterborne disease incidence and child mortality (children aged <2 y) since implementation of continuous supply, excluding boundary households

|  | Intermittent Supply | | |  | Continuous Supply | | | | | | | |
| --- | --- | --- | --- | --- | --- | --- | --- | --- | --- | --- | --- | --- |
|  | N | Number of HHs with case | I ^a^ |  | N | Number of HHs with case | I ^a^ | CIR | 95% CI ^b^ | Adjusted CIR ^c^ | 95% CI ^b^ | p-value ^d^ |
| Typhoid | 1690 | 103 | 60.9 |  | 1377 | 49 | 35.6 | 0.58 | (0.42–0.81) | 0.61 | (0.42–0.87) | 0.35 |
| Cholera | 1691 | 4 | 2.4 |  | 1378 | 5 | 3.6 | 1.53 | (0.37–7.36) | -- ^e^ | -- | 1.00 |
| Hepatitis | 1690 | 46 | 27.2 |  | 1377 | 50 | 36.3 | 1.33 | (0.91–1.98) | 1.22 | (0.81–1.88) | 0.35 |
| <2 y old child death | 1695 | 20 | 11.8 |  | 1378 | 9 | 6.5 | 0.55 | (0.22–1.15) | 0.48 | (0.17–1.04) | 0.13 |

Abbreviations: HH, household; I, incidence; CIR, cumulative incidence ratio; CI, confidence interval.

^a^ Households with at least one reported case (per 1,000 households) since implementation of continuous supply. ^b^ CIs obtained by bootstrapping within strata of wards. ^c^ Adjusted for household socioeconomic status, religion, handwashing infrastructure, latrine ownership, sewerage, and garbage disposal; we only included covariates in the adjusted models that could not plausibly be impacted by the continuous supply intervention. ^d^ p-value from Wilcoxon rank-sum permutation test; the permutation test is conservative relative to the CIs around the CIR because it tests the null hypothesis that the two groups have the same distribution as opposed to the null hypothesis of no effect on average. ^e^ Adjusted CIR not calculated because of sparse data.
