# Supplementary material for: Upgrading a Piped Water Supply from Intermittent to Continuous Delivery and Association with Waterborne Illness: A Matched Cohort Study in Urban India
Source: PLoS Med. 2015 Oct 27;12(10):e1001892. doi: 10.1371/journal.pmed.1001892 (PMC4624240; doi:10.1371/journal.pmed.1001892)
Supplement: S8 Table — (DOCX) [file pmed.1001892.s009.docx]

**S8 Table. Inverse probability of censoring-weighting analysis of child diarrheal illness outcomes**

|  |  |  |
| --- | --- | --- |
|  | Adjusted PR ^a^ | 95% CI ^b^ |
| HCGI | 1.01 | (0.92–1.11) |
| Diarrhea (primary outcome) | 0.92 | (0.82–1.04) |
| Blood or mucus in stool | 0.76 | (0.59–0.98) |

Abbreviations: PR, prevalence ratio; CI, confidence interval; HCGI, highly credible gastrointestinal illness.

^a^ Adjusted for child age, child sex, season, household socioeconomic status, religion, handwashing infrastructure, latrine ownership, sewerage, and garbage disposal; we only included covariates in the adjusted models that could not plausibly be impacted by the continuous supply intervention. ^b^ CIs obtained by weighted bootstrapping within strata of wards with clustering at household level, weights obtained from inverse probability of censoring.
