# Supplementary material for: Upgrading a Piped Water Supply from Intermittent to Continuous Delivery and Association with Waterborne Illness: A Matched Cohort Study in Urban India
Source: PLoS Med. 2015 Oct 27;12(10):e1001892. doi: 10.1371/journal.pmed.1001892 (PMC4624240; doi:10.1371/journal.pmed.1001892)
Supplement: S1 Text — (DOCX) [file pmed.1001892.s010.docx]

**S1 Text. Details of statistical methods**

*Participant Selection.* We used a genetic matching algorithm to select the control units (in this study, units = wards) to match the treated units that had been selected non-randomly by the local authorities prior to our evaluation. Genetic matching is a multivariate matching approach that uses an evolutionary search algorithm to efficiently find a set of matched pairs of treatment and control units that maximize the balance between the groups on observed characteristics [1]. The algorithm assigns weights to the covariates according to their importance in achieving balance, searches through distance metrics and chooses the metric and the weights that give the optimal overall covariate balance (measured by p-values from Kolmogorov-Smirnov tests and paired t-tests on the covariates that are matched on) [1]. We implemented the match using the *GenMatch* function of the *Matching* package in R [2]. We used the following variables to match the eight treatment (continuous supply) wards to eight control (intermittent supply) wards: percentages of *pukka*, low-income, one-room and slum households, percentage of illiterate females, percentages of households with own tap, receiving water less often than every five days, with own latrine and with a designated garbage disposal location provided by the municipality and garbage collection service, and monthly household health expenses. We evaluated the performance of the match by examining the pre-matching and post-matching standardized differences (difference between ward-level group means expressed as a percentage of the ward-level standard deviation in the treatment group) on all characteristics that contributed to the match [3].

*Effect Estimates and Inference*. We used generalized linear models to compare outcomes between study groups. We estimated standard errors and confidence intervals using a bootstrap with 5000 replications, stratified at the ward level to reflect the structure of the treatment/control units and clustered at the household level (i.e., resampling households with replacement within each ward) to account for repeated measures within children and multiple children per household. This approach defines the target population as the 16 study wards and limits the inference to this specific population rather than generalizing beyond the enrolled wards. To non-parametrically compare study groups and test the null hypothesis that the two groups have the same distribution, we generated ward-level means for all study outcomes and conducted permutation tests, stratified on matched ward-pairs, with a Wilcoxon rank-sum test statistic and 5000 replications [4].

*Subgroup Analyses.* We assessed effect modification by weather (rainy vs. dry) and household socio-economic status (SES). To characterize the SES of study households, we generated a wealth index using principal components analysis (PCA) [5]. We collected reported data on the ownership of: house, number of rooms, cabinet, table, chairs, tv, cd/dvd player, grinder, fridge, mobile phone, bicycle, motorbike, car, rickshaw, oxcart, any other vehicle and livestock. In addition, we observed housing materials (concrete vs. bamboo/mud). We performed PCA with these variables and used the first component to generate an asset score for each household [6]; the first component explained 23% of the variability in the original data. The asset score had an approximately Gaussian distribution, suggesting reasonable variation in SES in our study population and demonstrating that the variables included in the score adequately distinguished these variations. We split the study population into quartiles based on the asset score and defined the bottom two quartiles as below-median wealth. Tabulation of asset ownership in below- vs. above-median wealth households demonstrated good internal coherence; for example, 15% of above-median households owned a car compared to 0.1% of below-median households. Similarly, among above-median households 55% owned a fridge and 100% owned a grinder, compared to 2% and 70%, respectively, among below-median households. This binary variable (below- vs. above-median wealth) was used to generate an interaction term with the treatment variable (continuous vs. intermittent supply) to assess effect modification by SES on the multiplicative scale in the log-linear models. Similarly, a binary variable for rainy vs. dry weather based on whether a local weather station measured rain in the 10 days preceding the interview was used to generate an interaction term with the treatment variable to assess effect modification by rainfall status.

*Adjustment for Missing Data*. We conducted a complete-case analysis (84% of all enrolled), assuming that data were missing completely at random [7]. In addition, we used inverse probability weighting to adjust effect estimates for missingness as a robustness check; assuming that outcome data are missing at random, this method yields unbiased effect estimates for the original population (prior to loss to follow-up) by re-weighting data from households that remained in the study (thus “filling in” for households with similar characteristics that were lost) so that the study population characteristics are consistent with the enrolled cohort [8]. We used logistic regression to model the probability of a household completing the study as a function of household-level covariates measured during the first round of data collection including socioeconomic indicators (number of rooms, housing materials, assets, home ownership), demographic indicators (number of household members and <5 children, mother’s age and education, father’s employment status, religion) and sanitation and hygiene indicators (presence of handwashing station, latrine access, sewerage facilities). All covariates that were significant at the p=0.2 level in bivariate analyses were included in the multivariable model, and a final regression model was developed by backward stepwise elimination. In the final model, households that remained in the study had more household members and older mothers of <5 children, were more likely to own chairs, a tv, a bicycle and their own home and to have a handwashing facility in their home. The fitted model gives the predicted probability of remaining in the study as a function of these covariates. Households that completed the study were assigned weights equal to the inverse of their predicted probability of remaining in the study, and a weighted regression was conducted to adjust effect estimates for missingness, using generalized linear models with confidence intervals obtained with weighted bootstrapping.

1. Diamond A, Sekhon JS. Genetic matching for estimating causal effects: A general multivariate matching method for achieving balance in observational studies. Rev Econ Stat. 2013;95: 932–945.

2. Sekhon JS. Multivariate and Propensity Score Matching Software with Automated Balance Optimization: The Matching Package for R. J Stat Softw. 2011;42: 1–52.

3. Austin PC. Using the Standardized Difference to Compare the Prevalence of a Binary Variable Between Two Groups in Observational Research. Commun Stat - Simul Comput. 2009;38: 1228–1234. doi:10.1080/03610910902859574

4. Small DS, Ten Have TR, Rosenbaum PR. Randomization Inference in a Group–Randomized Trial of Treatments for Depression. J Am Stat Assoc. 2008;103: 271–279. doi:10.1198/016214507000000897

5. Vyas S, Kumaranayake L. Constructing socio-economic status indices: how to use principal components analysis. Health Policy Plan. 2006;21: 459.

6. Houweling TA, Kunst AE, Mackenbach JP. Measuring health inequality among children in developing countries: does the choice of the indicator of economic status matter? Int J Equity Health. 2003;2: 8. doi:10.1186/1475-9276-2-8

7. Wood AM, White IR, Thompson SG. Are missing outcome data adequately handled? A review of published randomized controlled trials in major medical journals. Clin Trials. 2004;1: 368–376. doi:10.1191/1740774504cn032oa

8. Hernán MA, Hernández-Díaz S, Robins JM. A Structural Approach to Selection Bias: Epidemiology. 2004;15: 615–625. doi:10.1097/01.ede.0000135174.63482.43
